# Quantum Computation Based on Photons with Three Degrees of Freedom

**DOI:** 10.1038/srep25977

**Published:** 2016-05-13

**Authors:** Ming-Xing Luo, Hui-Ran Li, Hong Lai, Xiaojun Wang

**Affiliations:** 1 Information Security and National Computing Grid Laboratory, Southwest Jiaotong University, Chengdu 610031, China; 2 School of Computer and Information Science, Southwest University, Chongqing 400715, China; 3 School of Electronic Engineering, Dublin City University, Dublin 9, Ireland

## Abstract

Quantum systems are important resources for quantum computer. Different from previous encoding forms using quantum systems with one degree of freedom (DoF) or two DoFs, we investigate the possibility of photon systems encoding with three DoFs consisting of the polarization DoF and two spatial DoFs. By exploring the optical circular birefringence induced by an NV center in a diamond embedded in the photonic crystal cavity, we propose several hybrid controlled-NOT (hybrid CNOT) gates operating on the two-photon or one-photon system. These hybrid CNOT gates show that three DoFs may be encoded as independent qubits without auxiliary DoFs. Our result provides a useful way to reduce quantum simulation resources by exploring complex quantum systems for quantum applications requiring large qubit systems.

Quantum computer has shown its superiority for solving difficult problems such as the large integer decomposition^1^,^2^,^3^ and data searching^4^,^5^. Since its difficult the large integer decomposition is the mathematical foundation of the well-known RSA cryptography which may be used for classical cryptographic applications^6^,^7^,^8^. Most of these quantum computation tasks may be completed with evolutions of quantum systems and desired quantum measurements^1^,^2^,^3^,^4^,^5^,^9^,^10^. If the quantum circuit model^11^ is applied, these evolutions may be synthesized by series of local quantum gates. Exactly, proper small gates such as the controlled phase-flip (CZ) gate or controlled-not (CNOT) gate combined with single-qubit gates^12^,^13^,^14^ can be used to implement quantum tasks with multiple qubits. These small gates construct a universal quantum gate set for quantum computing. Up to now, the CNOT gate has been widely implemented using several quantum systems, such as the linear optics^15^,^16^, ion trap^17^,^18^, atom^19^,^20^, and nuclear magnetic resonance^21^,^22^.

The solid-state quantum system has also attracted much attentions in quantum simulations because of its special optical property and scalability^23^,^24^,^25^,^26^. Moreover, electron-spin qubits associated with the nitrogen-vacancy (NV) defect centers are particularly useful. In fact, due to the long room-temperature coherent time^27^, the negatively charged NV defect center in the diamond lattice, consisting of a substitutional ^14^N atom and an adjacent vacancy, is an attractive candidate for quantum information processing. It has been used to prepare and detect optical sources^28^,^29^,^30^,^31^,^32^,^33^, generate hybrid quantum entanglements between the NV center and photon^34^, or electrons^35^,^36^, purify two-photonic hyperentanglement in both the polarization and spatial DoFs^37^, or implement the CZ gate between the NV centers assisted by the microsphere cavity^38^,^39^. The single-electron and nuclear-spin states can be faithfully detected even under ambient conditions^40^,^41^, when the electron spin of the NV defect center couples to nearby ^13^C nuclear spins. Another diamond NV^−^ center is proposed with six electrons from the nitrogen and three carbons surrounding the vacancy^42^, which is confined in a microtoroidal resonator (MTR)^43^ with the quantized whispering-gallery mode (WGM). This useful system allows for an ultrahigh-*Q* and a small mode volume of WGM microresonators^44^,^45^,^46^, which has been applied to construct quantum gates on electron-spin qubits^47^,^48^ or remote qubits^49^,^50^. Furthermore, recent experiments have assembled several hybrid systems, where colour centers in diamond nanocrystals or bulk diamond are coupled to the evanescent fields of cavities, which are defined in non-diamond materials coupling to WGMs in a silica micro-sphere^50^,^51^,^52^, diamond-GaP micro-disk^53^, GaP micro-ring cavities^54^, or SiN photonic crystal^55^.

Most of previous quantum simulations focused on systems with single DoF^15^,^16^,^17^,^18^,^19^,^20^,^21^,^22^ or hybrid systems^56^,^57^,^58^,^59^,^60^. A few schemes have considered photons with two DoFs^61^,^62^. Our recent result^63^ presents the independence of two DoFs (polarization DoF and spatial DoF) of photonic system, and then is used to construct the ququart (four-dimensional) quantum logic^64^. Thus quantum simulation resources may be saved one half. In this paper, we further reduce quantum resources by considering photonic systems with three DoFs. Motivated by recent schemes^65^,^66^, each photon may be encoded with two circularly polarized states and four modes, i.e., |*l, I*〉,|*r, I*〉 and |*l, E*〉,|*r, E*〉 for two crystal emissions. Here, *l*(*r*) refers to the left (right) side of each cone and *I*(*E*) denotes the internal (external) cone, as shown in Fig. 1. A general state is given by the product of one polarization state and two longitudinal momentum states. In the follow, we will investigate these photonic DoFs for the quantum simulation, without using auxiliary DoFs. From the quantum circuit model, CNOT gate will be schematically implemented on these DoFs of photonic states assisted by NV centers. For the symmetry of two spatial modes in each photonic system, fifteen CNOT gates are required to operate on the polarization DoFs and spatial-DoFs of the two-photon or one-photon system. Each gate is completed by interacting photons to auxiliary NV centers, disentangling NV centers, and correcting the emitting photons. These schemes are beyond to previous CNOT gates on the same DoF of two-photon state^14^,^15^,^56^,^57^, hybrid CNOT gates on the photon and stationary electron spins in quantum dots^58^,^59^,^60^,^61^, or different DoFs of two photons^62^,^63^. Our theoretical result shows that three DoFs of each photonic system can be used as independent qubits in one quantum task. Hence, two thirds of quantum resources may be saved for quantum simulations with large qubit systems, such as the Shor’s algorithm.

## Results

To show the encoding independence of the polarization DoF and two spatial DoFs of each photon, it is necessary to prove that all quantum transformations in *SU*(2^*n*^) may be implemented on these DoFs. Based on the theory of the universal logic gates^12^,^13^,^14^, it is sufficient to consider the CNOT gate on any two DoFs of the photonic system. It means that fifteen CNOT gates should be performed on photonic systems with three DoFs, where nine CNOT gates are on the two-photon system (all combinations of three DoFs) and six CNOT gates are on the one photon system. By exploring optical selection rules of the NV center in the crystal cavity, these CNOT gates may be realized without altering DoFs and auxiliary DoFs during implementations. In this case, each photonic DoF can be encoded as an independent qubit in quantum applications.

### Photon with three DoFs

**Figure 1:**
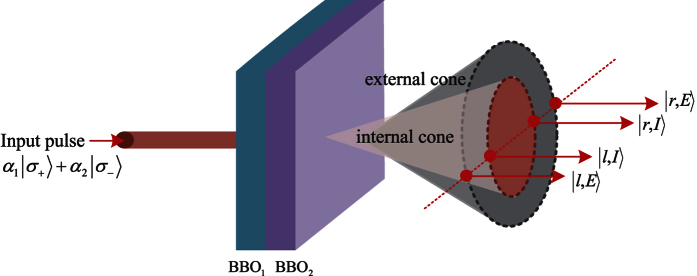
Schematic photon generation from the internal (*I*) and external (*E*) cone assisted by a two-crystal system. Polarized state as an input pulse passes through two 0.5 mm thick Type I *β*-barium-borate (BBO) crystal slabs. The input photon is created at a degenerate wavelength *λ* = 2*λ*_*p*_ by each BBO crystal along two correlated directions belonging to the lateral surfaces of two SPDC cones, with full aperture angles *θ*_*I*_ and *θ*_*E*_, respectively. The internal (*I*) and external (*E*) cone correspond to the first and the second crystal, respectively. The annular sections of each emission cone, with approximate diameters *d*_*I*_ and *d*_*E*_ are intercepted by a single eight-hole screen. The dichotomy existing between the *I* cone and *E* cone is identified as an independent DoF. The corresponding mode emission as *l*(*r*) by referring to the left (right) side of each cone.

Circularly polarized photon in the state *α*_1_|*L*〉 + *α*_2_|*R*〉 (left circularly polarized state |*L*〉 and right circularly polarized state |*R*〉) is created at a degenerate wavelength *λ* = 2*λ*_*p*_ by each BBO crystal along two correlated directions belonging to the lateral surfaces of two SPDC cones, with full aperture angles *θ*_*I*_ and *θ*_*E*_, respectively^64^,^65^, as shown in Fig. 1. The output state is dependent of these angles. *I* refers to the internal cone whereas *E* denotes the external cone, corresponding to the first and the second crystal, respectively. The dichotomy existing between the *I* cone and *E* cone is thus identified as an independent DoF, i.e., the corresponding mode emission as *l*(*r*) by referring to the left (right) side of each cone^64^,^65^. If the pump coherence length exceeds more than one order of magnitude the total crystal length, the coherence and indistinguishability between two crystal emissions may be guaranteed^64^,^65^. Two conical emissions are then transformed into two cylindrical ones by a positive lens with focal length *f*, located at a distance *f* from the intermediate point of the second crystal device. By selecting four pairs of correlated modes with an eight-hole screen, |*l, I*〉 and |*r, I*〉 for the first crystal and |*l, E*〉 and |*r, E*〉 for the second crystal emission, a general photonic state is prepared as the product of one polarization state and two longitudinal momentum states (or, equivalently, a ququart state) and is expressed as a 3-qubit state:


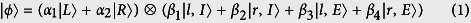


where |*α*_1_|^2^ + |*α*_2_|^2^ = 1 and |*β*_1_|^2^ + |*β*_2_|^2^ + |*β*_3_|^2^ + |*β*_4_|^2^ = 1. Here, *β*_*j*_ are dependent of aperture angles *θ*_*I*_ and *θ*_*E*_, and focal length *f*, which are not goals in this paper^64^,^65^.

### A diamond NV center coupled to an MTR with a WGM

**Figure 2:**
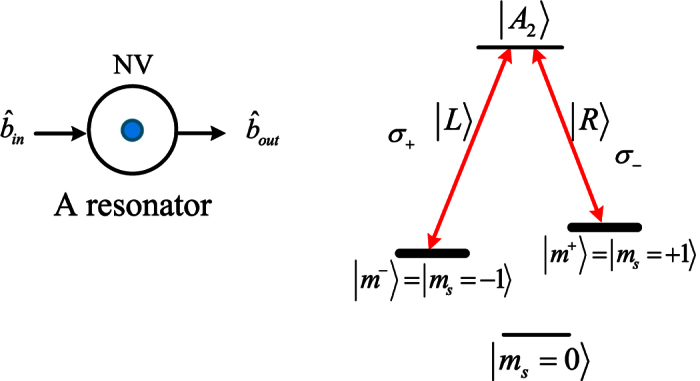
Schematic NV center coupling to the resonator and possible Λ-type optical transitions in the NV center. 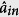
 and 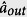 are the input and output field operators of a waveguide, respectively. The bold levels encode qubits, i.e., |*m*^±^〉 = |*m*_*s*_ = ±〉. The transition |*m*^−^〉 → |*A*_2_〉 is derived by a left circularly polarized photon *σ*_+_ (|*L*〉), and |*m*^+^〉 → |*A*_2_〉 is derived by a right circularly polarized photon *σ*_−_ (|*R*〉).

Schematic NV center in a diamond embedded in a photonic crystal cavity is shown in Fig. 2. The negatively charged NV center is consisted of a substitutional nitrogen atom and an adjacent vacancy with six electrons. The Λ-type three-level system is realized using specific excited state |*A*_2_〉 = (|*E*_−_〉|*m*^+^〉 + |*E*_+_〉|*m*^−^〉) as an ancillary state^66^,^34^. Here, |*E*_±_〉 are orbital states with angular momentum projection along the NV axis. The ground state is an electronic spin triplet with a splitting of 2.88 GHz between the magnetic sublevels |0〉(*m*_*s*_ = 0) and |*m*^±^〉(*m*_*s*_ = ±1)^34^. |*A*_2_〉 may decay into two ground states |*m*^−^〉 and |*m*^+^〉 by exciting the NV center with a polarized 2-ns *p*-pulse that is shorter than the emission timescale, and the reflection may be separated from fluorescence photons using detection timing^34^. The normal boundary condition 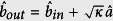 is used to derive the optical selection rule with the input field 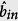, output field 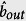 and cavity field operator 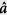. If spins stay in the ground states most of the time^67^, the optical reflection coefficient may be approximately defined in the follow (see Methods)


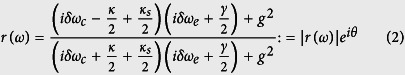


where *δω*_*c*_ and *δω*_*e*_ are frequency detunings satisfying *δω*_*c*_ = *ω*_*c*_ − *ω* and *δω*_*e*_ = *ω*_*e*_ − *ω. ω*_*c*_, *ω* and *ω*_*e*_ are the frequencies of the cavity mode, input photon pulse, and NV center, respectively. *g* is the coupling strength between the cavity and the NV center. *κ, κ*_*s*_ and *γ* are the damping rate of the cavity, cavity side leakage mode, and spontaneous decay rate of the NV center, respectively. If define the cooperativity *C* = 2*g*^2^/(*γκ*), the photonic reflection probability^68^ is determined by the cooperativity *C* and the cavity tuning as follow


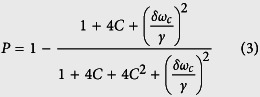


Considering the coupling strength *g* = 0, an NV center is uncoupled from the cavity (the cold cavity), and the reflection coefficient *r*(*ω*) becomes


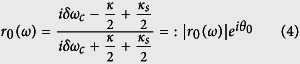


Thus the input pulse in the polarized state |*L*〉 gains a phase shift *θ* after reflecting from the hot cavity (*g* > 0) with the NV center |*m*^−^〉, or a phase shift *θ*_0_ after reflecting from the cold cavity (*g* = 0) with the NV center |*m*^+^〉. The input pulse in the state |*R*〉 gains a phase shift *θ*_0_ after reflecting from the cold cavity with the NV center |*m*^−^〉, or a phase shift *θ* after reflecting from the hot cavity with the NV center |*m*^+^〉. By choosing a proper frequency detuning *δω*_*e*_ = 0^66^ and the cooperativity *C* ≫ 1, the reflection coefficients may satisfy |*r*(*ω*)| ≈ 1 and |*r*_0_(*ω*)| ≈ 1 when the cavity side leakage *κ*_*s*_ is negligible. By adjusting the frequencies *ω* and *ω*_*c*_ such that *δω*_*c*_/*κ* → 0 and *C* ≫ 1, the phase shifts may be realized as *θ* = 0 and *θ*_0_ = *π*. Hence, the following optical transition may be obtained as


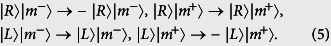


From this optical transition, an NV center requires a polarization-degenerate cavity mode, which is also suitable in H1 photonic crystals^69^,^70^ and fiber-based cavities^71^.

### CNOT gate on the same DoF of the two-photon system

**Figure 3:**
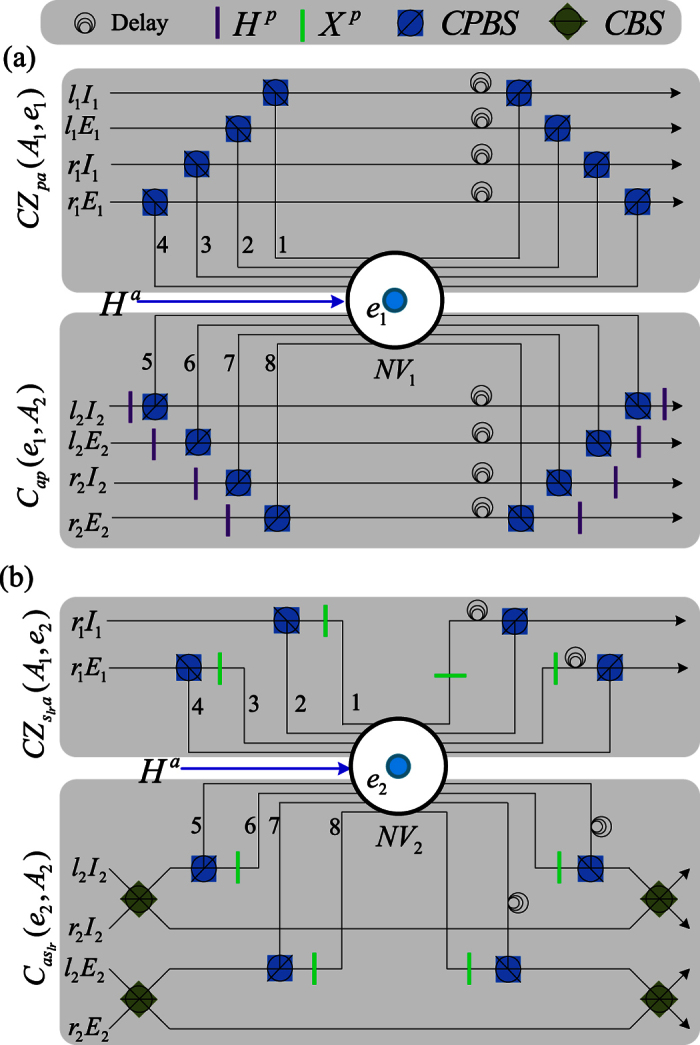
Schematic CNOT gate on the same DoF of two photons. (**a**) Schematic CNOT gate on the polarization DoFs of two photons. CPBS represents a polarizing beam splitter in the circular basis, which transmits |*R*〉 and reflects |*L*〉. CBS represents a 50% 50 beam splitter to perform the Hadamard operation on the spatial DoF of a photon. *X* represents a waveplate to implement the bit-flip operation *X*^*p*^ = |*R*〉〈*L*| + |*L*〉〈*R*|. *H*^*a*^ represents the Hadamard operation on the NV center in a cavity. (**b**) Schematic CNOT gate on the spatial DoFs of two photons. *H*^*p*^ represents a half-wave plate (HWP) to perform the Hadamard operation on the polarization DoF of a photon. The numbers 1, 2, …, 8 denote the orders for an input pulse to interact with an NV center.

Schematic CNOT gate on the same DoF of the two-photon system is shown in Fig. 3. NV centers *e*_*i*_ trapped in the photonic crystal NV_*i*_ are initially prepared in the superposition states 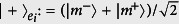. Two input photons *A*_1_ and *A*_2_ are in the states 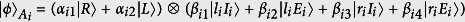, *i* = 1, 2. Figure 3(a) is used to complete the CNOT gate on the polarization DoFs of two photons, i.e.,


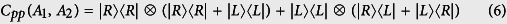


In detail, the photon *A*_1_ from each spatial mode (*l*_1_*I*_1_, *l*_1_*E*_1_, *r*_1_*I*_1_ or *r*_1_*E*_1_) evolves as *CPBS* → *NV*_1_ → *CPBS* to complete the following controlled phase gate


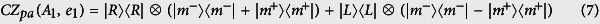


on the polarization DoF and the NV center *e*_1_ (see Appendix A of Supplementary Information for details). And then, after one Hadamard operation *H*^*a*^ on the NV center *e*_1_ in the NV_1_, the photon *A*_2_ from each spatial mode evolves as *H*^*p*^ → *CPBS* → *NV*_1_ → *CPBS* → *H*^*p*^ to complete the following hybrid CNOT gate


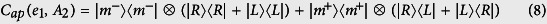


on the NV center *e*_1_ and the polarization DoF of the photon *A*_2_ (see Appendix A of Supplementary Information for details). Now, after disentangling the NV center *e*_1_ using the measurement under the basis 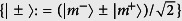, the phase flip *Z*^*p*^ = |*R*〉〈*R*| − |*L*〉〈*L*| is performed on the photon *A*_1_ from each mode for the measurement outcome 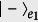. Thus the CNOT gate *C*_*pp*_(*A*_1_, *A*_2_) has been realized on the photons *A*_1_ and *A*_2_.

Figure 3(b) is a schematic circuit to complete the CNOT gate on the spatial DoF {*l, r*}s of two photons, i.e.,


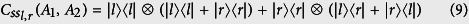


Here, the photon *A*_1_ from each spatial mode *r*_1_*I*_1_ or *r*_1_*E*_1_ evolves as *CPBS* → *NV*_2_ → (*X* → *NV*_2_ → *X*) → *CPBS* to complete the following controlled phase gate


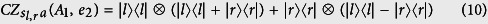


on the spatial DoF {*l, r*} and the NV center *e*_2_ in the state |+〉 (see Appendix B of Supplementary Information for details). Now, after a Hadamard gate *H*^*a*^ performed on the NV center *e*_2_ in the NV_2_, the followed circuit *CBS* → *CPBS* → *NV*_2_ → (*X* → *NV*_2_ → *X*) → *CPBS* → *CBS* for each mode pair (*l*_2_*I*_2_, *r*_2_*I*_2_) or (*l*_2_*E*_2_, *r*_2_*E*_2_) is used to complete the hybrid CNOT gate on the NV center *e*_2_ and the spatial DoF {*l, r*} of the photon *A*_2_ (see Supplementary Information for details), i.e.,


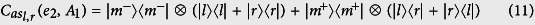


Now, the CNOT gate 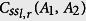 may be realized by disentangling the NV center *e*_2_ using the measurement under the basis {|±〉}, where *Z*^*p*^ is performed on the photon *A*_1_ from each spatial mode *r*_1_*I*_1_ and *r*_1_*E*_1_ for the measurement outcome 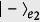.

A similar CNOT gate


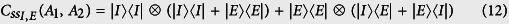


holds for the spatial DoF {*I, E*}s of two photons using an NV center *e*_3_ trapped in the optical cavity NV_3_ (see Appendix C of Supplementary Information for details).

### Hybrid CNOT gate on the different DoFs of the two-photon system

**Figure 4:**
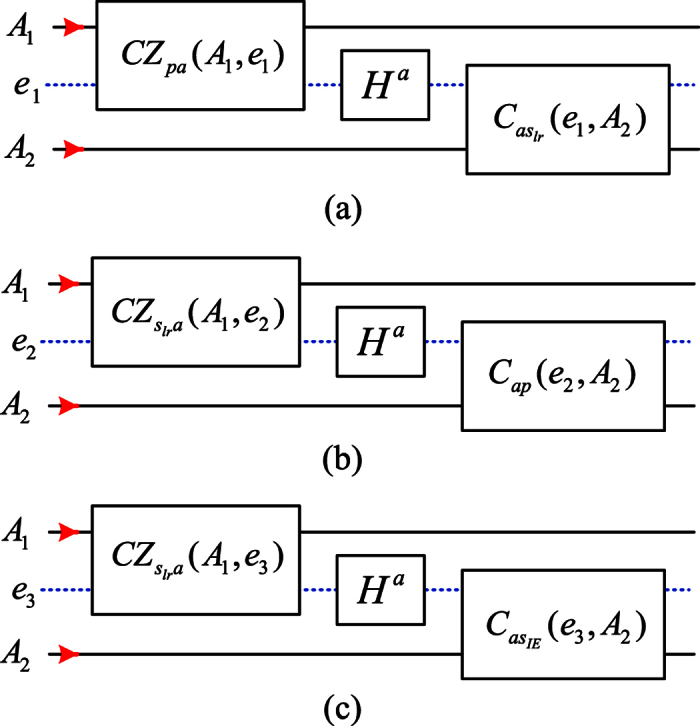
Schematic CNOT gate on different DoFs of the two-photon system. (**a**) Schematic CNOT gate on the polarization DoF of one photon and the spatial DoF {*l, r*} of the other. (**b**) Schematic CNOT gate on the spatial DoF {*l, r*} of one photon and the polarization DoF of the other. (**c**) Schematic CNOT gate on the spatial DoF {*l, r*} of one photon and the spatial DoF {*I, E*} of the other photon. *e*_*i*_ denote auxiliary NV centers in the NV-cavity *NV*_*i*_, *i* = 1, 2, 3. The subcircuits *H*^*a*^, *CZ*_*pa*_, *C*_*ap*_, *CZ*_*sa*_ and *C*_*as*_ are shown in the Fig. 3.

Figure 4(a) is a schematic circuit to implement the CNOT gate on the polarization DoF of the photon *A*_1_ and the spatial DoF {*l, r*} of the photon *A*_2_, i.e.,


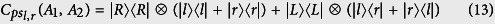


In fact, similar to the Fig. 3(a), the first controlled phase flip *CZ*_*pa*_(*A*_1_, *e*_1_) in the equation (7) is used to change the photon *A*_1_ and the NV center *e*_1_ from 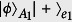 to 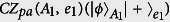. And then, after one Hadamard operation *H*^*a*^ performed on the NV center *e*_1_ in the NV_1_, the followed circuit *CBS* → *CPBS* → *NV*_1_ → (*X* → *NV*_1_ → *X*) → *CPBS* → *CBS* for each spatial mode pair (*l*_2_*I*_2_, *r*_2_*I*_2_) or (*l*_2_*E*_2_, *r*_2_*E*_2_) is used to complete the CNOT gate *C*_*as*_(*e*_2_, *A*_1_) in the equation (11) on the NV center *e*_1_ and the spatial mode {*l, r*} of the photon *A*_2_ (similar to the Fig. 3(b)). After disentangling the NV center *e*_1_ using the measurement under the basis {|±〉}, the hybrid CNOT gate 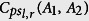 is realized on the photons *A*_1_ and *A*_2_, where *Z*^*p*^ is performed on the photon *A*_1_ from each spatial mode *r*_1_*I*_1_ and *r*_1_*E*_1_ for the measurement outcome 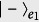, see Appendix D of Supplementary Information for details.

Similarly, after the controlled-phase flip *CZ*_*pa*_(*A*_1_, 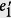) on the photon *A*_1_ and the NV center *e*_1′_ in the state |+〉, a schematic circuit is applied to the photon *A*_2_ from two spatial mode pairs (*l*_2_*I*_2_, *l*_2_*E*_2_) and (*r*_2_*I*_2_, *r*_2_*E*_2_) to complete the CNOT gate on the NV center *e*_1′_ and the spatial DoF {*I, E*} of the photon *A*_2_ (see Appendix E of Supplementary Information for details). The hybrid CNOT gate


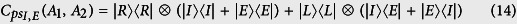


is implemented on the polarization DoF of the photon *A*_1_ and the spatial DoF {*I, E*} of the photon *A*_2_ after disentangling the NV center *e*_1′_.

Figure 4(b) is used to implement the CNOT gate on the spatial DoF {*l, r*} of the photon *A*_1_ and the polarization DoF of the photon *A*_2_, i.e.,


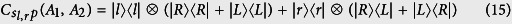


In fact, similar to the evolutions as shown in the Fig. 3(b), the controlled phase gate 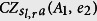 in the equation (10) is performed on the photon *A* and the NV center *e*_2_ in the state |+〉 to get 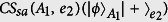. And then, after one Hadamard operation *H*^*a*^ on the NV center *e*_2_ in the NV_2_, the followed circuit for the photon *A*_2_ from each spatial mode is used to complete the CNOT gate *C*_*ap*_(*e*_2_, *A*_2_) on the NV center *e*_2_ and the polarization DoF of the photon *A*_2_ (see the Fig. 3(a)). The final joint state is 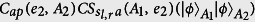. Finally, by disentangling the NV center *e*_2_ using the measurement under the basis {|±〉}, the hybrid CNOT gate 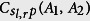 is realized, where −*I*^*p*^ will be performed on the photon *A*_1_ from each spatial mode *r*_1_*I*_1_ and *r*_1_*E*_1_ for the measurement outcome 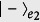, see Appendix F of Supplementary Information for details. Moreover, if the second part of the present circuit above is applied to the photon *A*_1_ from two spatial modes *l*_1_*I*_1_ and *l*_1_*E*_1_, the CNOT gate is implemented on the spatial DoF {*I, E*} of the photon *A*_1_ and the polarization DoF of the photon *A*_2_, see Appendix G of Supplementary Information for details.

Figure 4(c) is used to implement the CNOT gate on the spatial DoF {*l, r*} of the photon *A*_1_ and the spatial DoF {*I, E*} of the photon *A*_2_, i.e,


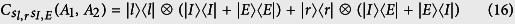


In detail, similar to the evolutions as shown in the Fig. 3(b), the controlled phase gate 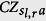 in the equation (10) is performed for the photon *A* and the NV center *e*_3_ in the state |+〉 to get 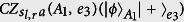. And then, after one Hadamard operation *H*^*a*^ on the NV center *e*_3_, the followed circuit for the photon *A*_2_ from each spatial mode is used to realized the CNOT gate 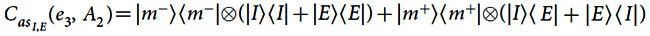 on the NV center *e*_3_ and the spatial DoF {*I, E*} of the photon *A*_2_. The final joint state is 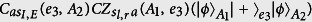. Now, by disentangling the NV center *e*_3_ using the measurement under the basis {|±〉}, 
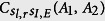
 may be deterministically realized, where −*I*^*p*^ will be performed on the photon *A*_1_ from each spatial mode *l*_1_*E*_1_ and *r*_1_*E*_1_ for the measurement outcome 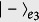, see Appendix H of Supplementary Information for details. Similarly, the CNOT gate may be implemented on the spatial DoF {*I, E*} of the photon *A*_1_ and the spatial DoF {*l, r*} of the photon *A*_2_, see Appendix I of Supplementary Information for details.

### Hybrid CNOT gate on different DoFs of one photon

**Figure 5:**
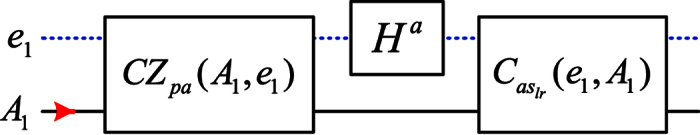
Hybrid CNOT gate on different DoFs of one photon. *e*_1_ denotes an auxiliary NV center in the NV-cavity *NV*_1_. The subcircuits *CZ*_*pa*_ and *C*_*as*_ are shown in the Fig. 3.

Figure 5 is a schematic circuit to implement the CNOT gate 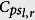 in the equation (13) on the polarization DoF and the spatial DoF {*l, r*} of the photon *A*_1_. In detail, similar to the Fig. 3(a), the controlled-phase flip *CZ*_*pa*_ in the equation (7) is used to change the photon *A*_1_ and the NV center *e*_1_ from 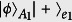 to 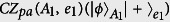. And then, after one Hadamard operation *H*^*a*^ performed on the NV center *e*_1_, the followed CNOT gate *C*_*asl*_ in the equation (11) is performed on the NV center *e*_1_ and the spatial DoF {*l, r*} of the photon *A*_1_ (similar to the Fig. 3(b)). After disentangling the NV center *e*_1_ using the measurement under the basis {|±〉}, the hybrid CNOT gate 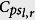 is realized on the photon *A*_1_, where *Z*^*p*^ will be performed for the photon *A*_1_ from each mode for the measurement outcome 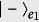, see Appendix J of Supplementary Information for details. Moreover, if the CNOT gate 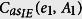 is performed on the NV center *e*_1_ and the photon *A*_1_ after *CZ*_*pa*_(*A*_1_, *e*_1_), the hybrid CNOT gate 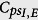 in the equation (14) is realized on the photon *A*_1_ after properly disentangling the auxiliary NV center, see Appendix K of Supplementary Information for details.

For the hybrid CNOT gate on the spatial DoF {*l, r*} and the spatial DoF {*I, E*} of the photon *A*_1_, the photon *A*_1_ from the spatial modes *r*_1_*I*_1_ and *r*_1_*E*_1_ passes through CBS, −*I*, CBS, sequentially. The photon *A*_1_ evolves as follows


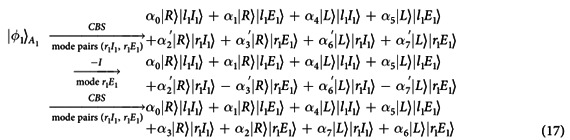


where 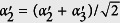, 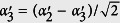, 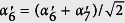, and 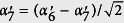. Similar circuit may be used to realize the hybrid CNOT gate on the spatial DoF {*I, E*} and the spatial DoF {*l, r*} of the photon *A*_1_, see Appendix K of Supplementary Information for details. Moreover, the CNOT gates on the spatial mode DoF and the polarization DoF of the photon *A*_1_ are easily realized by two flip waveplates on two spatial modes *r*_1_*I*_1_ and *r*_1_*E*_1_, or *l*_1_*E*_1_ and *r*_1_*E*_1_, respectively.

## Discussions

In ideal conditions, one may neglect the cavity side leakage, and the reflection coefficients satisfy |*r*_0_(*ω*)| ≈ 1 and |*r*(*ω*)| ≈ 1. The corresponding fidelities of the present CNOT gates are nearly 100%. In experiment, the general fidelity is defined by 
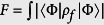
, where |Φ〉 is the ideal final state without side leakage while *ρ*_*f*_ is the final state under a real situation with side leakage. In the resonant condition *δω*_*e*_ = 0, if the cavity side leakage is considered, the optical selection rule for the NV-cavity system given by the equation (5) becomes


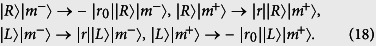


**Figure 6:**
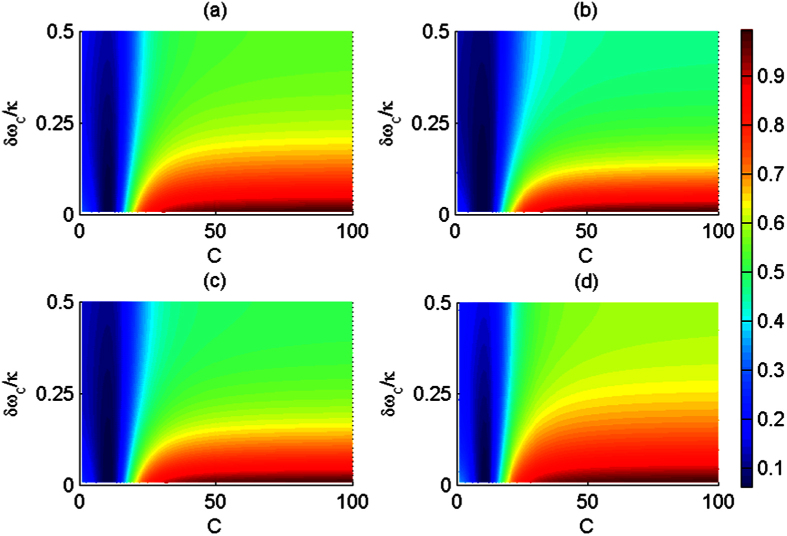
Average fidelities of the present CNOT gates vias the cooperativity *C* and relative detuning *δω*_*c*_/*κ*. (**a**) The average fidelity of the CNOT gate on the polarization DoFs of two photons; (**b**) The average fidelity of the CNOT gate on the spatial DoFs of two photons; (**c**) The average fidelity of the hybrid CNOT gate on the polarization and spatial DoFs of the two-photon system; (**d**) The average fidelity of the hybrid CNOT gate on the polarization and spatial DoFs of the one photon system. The average fidelity is computed as the expectation of random input photons.

**Figure 7:**
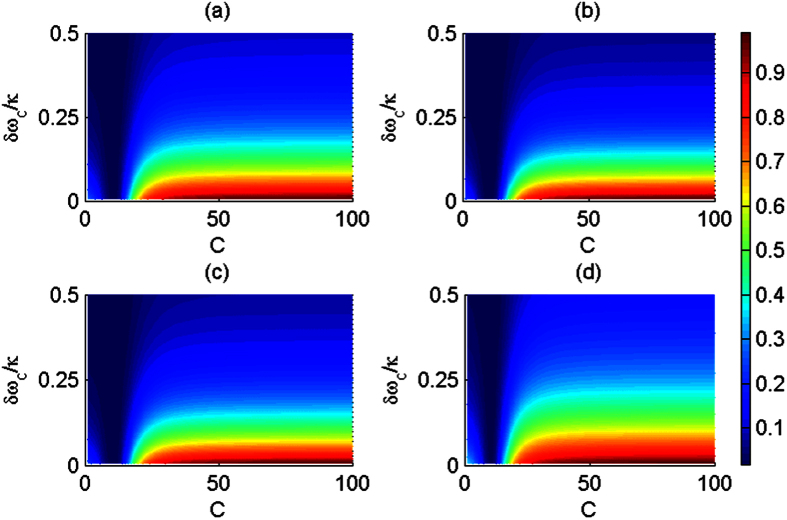
Average efficiencies of the present CNOT gates vias the cooperativity *C* and relative detuning *δω*_*c*_/*κ*. (**a**) The average fidelity of the CNOT gate on the polarization DoFs of two photons; (**b**) The average fidelity of the CNOT gate on the spatial DoFs of two photons; (**c**) The average fidelity of the hybrid CNOT gate on the polarization and spatial DoFs of the two-photon system; (**d**) The average fidelity of the hybrid CNOT gate on the polarization and spatial DoFs of the one photon system. The average fidelity is computed as the expectation of random input photons.

Due to the exchangeability of two spatial DoFs of one photon with respect to random initial photons, the fidelities and efficiencies are evaluated for four CNOT gates: CNOT gate on two polarization DoFs, CNOT gate on two spatial DoFs, CNOT gate on the polarization DoF of one photon and the spatial DoF of the other photon, and CNOT gate on the polarization and spatial DoFs of one photon system, as shown in Figs 6 and 7, respectively. Generally, large cooperativity *C* and low relative detuning *δω*_*c*_/*κ* are required for high fidelities and efficiencies. For the diamond NV centers, the photoluminescence is partially unpolarized, and the emission with ZPL is only 4% of the total emission. ZPL with zero phonon line is only 4% of *γ* = 2 × 15 MHz^31^. For the diamond NV center in a MTR with WGM mode system, |*r*(*ω*)| ≈ 0.95 when *C* ≥ 18^34^ with small detuning *δω*_*c*_/*κ* ≈ 0; |*r*(*ω*)| ≈ 1 when *C* ≥ 50 with small detuning *δω*_*c*_/*κ* ≈ 0 for *κ* ≈ 1 GHz or *κ* ≈ 10 GHz. For our CNOT gates, if *C* ≥ 18 and *δω*_*c*_/*κ* ≈ 0.1, their fidelities are greater than 82.6% and their efficiencies are greater than 75.4%. If *C* ≥ 50 and *δω*_*c*_/*κ* ≈ 0.1, their fidelities are greater than 98.4% and their efficiencies are greater than 94.7%.

In conclusion, we have investigated the possibility of quantum simulations using photon systems with three DoFs. We have constructed fifteen schematic CNOT gates operating on the spatial and polarization DoFs of the two-photon system or one-photon system. Different from previous CNOT gate on the same DoF of the two-photon system^14^,^15^,^56^,^57^, our schemes are based on different DoFs of two photons or one photon. Compared with hybrid implementations on the photon and stationary electron spins in quantum dots^58^,^59^,^60^,^61^, the present CNOT circuits are ultimately realized on the photon system, and the electron spins in NV center are auxiliary resources to build the correlation between photons. The present schemes have shown that two different spatial DoFs may be viewed as independent qubits simultaneously, which has beyond previous independence of the polarization and spatial DoFs^62^,^63^. Although different DoFs may be easily exchanged in terms of encoding, the schematic operations are inconvenient for photon systems with two different spatial DoFs. The main reason is that the hybrid CNOT gates are not realized in one-shot manners. Thus, it is difficult to exchange these DoFs during applications, where different DoFs may be used as different encoding types such as the quantum Shor algorithm or the quantum search algorithm. Hence, our results are distinct from all previous quantum logic gates on different photons^14^,^15^,^56^,^57^. Our theoretical schemes have shown that three DoFs of photon systems may be independent in quantum information processing. Two thirds of the quantum resources may be saved in quantum simulations. With the recent experiments of the NV-cavity system^33^,^34^,^35^, our schemes are expected to be applicable for the entanglement distribution or large-scale quantum computation.

## Methods

### A diamond NV center coupled to an MTR with a WGM

The master equation of the whole system may be expressed by a Lindblad form as follows


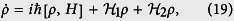


where *H* = *H*_1_ + *H*_2_ + *H*_3_ + *H*_4_. 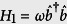 is the Hamiltonian of an input photon pulse. 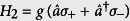 is the standard Jaynes-Cummings Hamiltonian for a two-level system interacting with a single electromagnetic mode by applying the rotating wave approximation and dropping the energy nonconserving terms. *σ*_−_ and *σ*_+_ are the Pauli raising and lowering operators, respectively. *g* is the coupling strength between the cavity and *X*^−^. 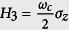 is the Hamiltonian of the dipole. *σ*_*z*_ is the Pauli operator for the population inversion. 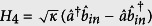 is the interaction between the excitation field and system. 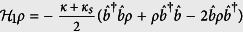 accounts for the damping of the input photon pulse. 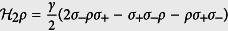 accounts for spontaneous emission of the dipole. The input-output optical relation of the NV center system may be calculated from the Heisenberg equations^67^ in terms of the cavity field operator 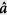, input pulse field 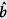 and dipole operator *σ*_−_,


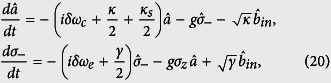


If spins stay in the ground states most of the time [〈*σ*_−_〉 = −1], the cavity output 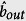 is connected with the input field by the standard input-output relation by a reflection coefficient *r*(*ω*).

### Measurement of the NV center *e* in cavity

To measure the NV center *e* of an entangled system *α*|*m*^−^〉_*e*_|Ω_1_〉 + *β*|*m*^+^〉_*e*_|Ω_2_〉, an auxiliary photon *c* in the state 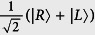 may be used as follows. Let the photon *c* pass through one CPBS to split the circular polarizations |*R*〉 and |*L*〉, and the right-circular polarization |*R*〉 interact with the cavity system, and its output combine with |*L*〉 of the photon *c* using the other CPBS. Thus, the joint system evolves


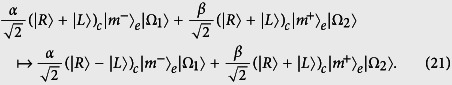


Hence, the NV center *e* can be determined by measuring the photon in the orthogonal basis 
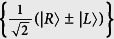
. The NV center is |*m*^−^〉 or |*m*^+^〉 for the measurement outcome 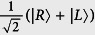 or 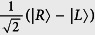, respectively.

## Additional Information

**How to cite this article**: Luo, M.-X. *et al*. Quantum Computation Based on Photons with Three Degrees of Freedom. *Sci. Rep.* **6**, 25977; doi: 10.1038/srep25977 (2016).

## Supplementary Material

Supplementary Information
