# Supplementary material for: Quantum Computation Based on Photons with Three Degrees of Freedom
Source: Sci Rep. 2016 May 13;6:25977. doi: 10.1038/srep25977 (PMC4865807; doi:10.1038/srep25977)
Supplement: Supplementary Information [file srep25977-s1.pdf]

Quantum Computation Based on Photons with Three Degrees of Freedom

Ming-Xing Luo[1], Hui-Ran Li[1], Hong Lai[2] & Xiaojun Wang [3]

[1] Information Security and National Computing Grid Laboratory,

Southwest Jiaotong University, Chengdu 610031, China,

[2] School of Computer and Information Science, Southwest University, Chongqing 400715, China,

[3] School of Electronic Engineering, Dublin City University, Dublin 9, Ireland

April 13, 2016

In this supplementary information, for the simplicity denote two input systems as

$$
\begin{aligned}
|\phi\rangle_{A_1} &= (\alpha_{11}|R\rangle+\alpha_{12}|L\rangle)\otimes(\beta_{11}|l_1I_1\rangle+\beta_{12}|l_1E_1\rangle+\beta_{13}|r_1I_1\rangle+\beta_{14}|r_1E_1\rangle)\\
&:= \alpha_0|R\rangle|l_1I_1\rangle+\alpha_1|R\rangle|l_1E_1\rangle+\alpha_2|R\rangle|r_1I_1\rangle+\alpha_3|R\rangle|r_1E_1\rangle\\
&\quad +\alpha_4|L\rangle|l_1I_1\rangle+\alpha_5|L\rangle|l_1E_1\rangle+\alpha_6|L\rangle|r_1I_1\rangle+\alpha_7|L\rangle|r_1E_1\rangle, \qquad (1)\\
|\phi\rangle_{A_2} &= (\alpha_{21}|R\rangle+\alpha_{22}|L\rangle)\otimes(\beta_{21}|l_2I_2\rangle+\beta_{22}|l_2E_2\rangle+\beta_{23}|r_2I_2\rangle+\beta_{24}|r_2E_2\rangle)\\
&:= \beta_0|R\rangle|l_2I_2\rangle+\beta_1|R\rangle|l_2E_2\rangle+\beta_2|R\rangle|r_2I_2\rangle+\beta_3|R\rangle|r_2E_2\rangle\\
&\quad +\beta_4|L\rangle|l_2I_2\rangle+\beta_5|L\rangle|l_2E_2\rangle+\beta_6|L\rangle|r_2I_2\rangle+\beta_7|L\rangle|r_2E_2\rangle. \qquad (2)
\end{aligned}
$$

Appendix A. CNOT gate on the polarization DoFs of two photons

First, from the Figure 3(a), the photon A_1 evolves as follows

$$
\begin{aligned}
|\phi\rangle_{A_1}|+\rangle_{e_1} &\xrightarrow[\text{path 1}]{CPBS,NV_1,CPBS} (\alpha_0|R\rangle|l_1I_1\rangle+\alpha_1|R\rangle|l_1E_1\rangle+\alpha_2|R\rangle|r_1I_1\rangle+\alpha_3|R\rangle|r_1E_1\rangle)|+\rangle_{e_1}\\
&\quad +\alpha_4|L\rangle|l_1I_1\rangle|-\rangle_{e_1}+(\alpha_5|L\rangle|l_1E_1\rangle+\alpha_6|L\rangle|r_1I_1\rangle+\alpha_7|L\rangle|r_1E_1\rangle)|+\rangle_{e_1}\\
&\xrightarrow[\text{path 2}]{CPBS,NV_1,CPBS} (\alpha_0|R\rangle|l_1I_1\rangle+\alpha_1|R\rangle|l_1E_1\rangle+\alpha_2|R\rangle|r_1I_1\rangle+\alpha_3|R\rangle|r_1E_1\rangle)|+\rangle_{e_1}\\
&\quad +(\alpha_4|L\rangle|l_1I_1\rangle+\alpha_5|L\rangle|l_1E_1\rangle)|-\rangle_{e_1}+(\alpha_6|L\rangle|r_1I_1\rangle+\alpha_7|L\rangle|r_1E_1\rangle)|+\rangle_{e_1}\\
&\xrightarrow[\text{path 3}]{CPBS,NV_1,CPBS} (\alpha_0|R\rangle|l_1I_1\rangle+\alpha_1|R\rangle|l_1E_1\rangle+\alpha_2|R\rangle|r_1I_1\rangle+\alpha_3|R\rangle|r_1E_1\rangle)|+\rangle_{e_1}\\
&\quad +(\alpha_4|L\rangle|l_1I_1\rangle+\alpha_5|L\rangle|l_1E_1\rangle+\alpha_6|L\rangle|r_1I_1\rangle)|-\rangle_{e_1}+\alpha_7|L\rangle|r_1E_1\rangle|+\rangle_{e_1}\\
&\xrightarrow[\text{path 4}]{CPBS,NV_1,CPBS} (\alpha_0|R\rangle|l_1I_1\rangle+\alpha_1|R\rangle|l_1E_1\rangle+\alpha_2|R\rangle|r_1I_1\rangle+\alpha_3|R\rangle|r_1E_1\rangle)|+\rangle_{e_1}\\
&\quad +(\alpha_4|L\rangle|l_1I_1\rangle+\alpha_5|L\rangle|l_1E_1\rangle+\alpha_6|L\rangle|r_1I_1\rangle+\alpha_7|L\rangle|r_1E_1\rangle)|-\rangle_{e_1} := |\Phi_1\rangle_{A_1e_1}
\end{aligned}
\qquad (3)
$$

This is a controlled-Z gate($CZ_{pe}(A_1, e_1)$) on the polarization state of the photon A_1 and the NV center e_1 [the NV center e_1 as the target qubit], i.e.,

$$CZ_{pe} := |R\rangle\langle R| \otimes (|m^-\rangle\langle m^-| + |m^+\rangle\langle m^+|) + |L\rangle\langle L| \otimes (|m^-\rangle\langle m^-| - |m^+\rangle\langle m^+|)$$

And then, after a Hadamard transformation H^a being performed on the NV center e_1, the photon A_2 evolves as follows

$$\begin{aligned}
|\Phi_1\rangle_{A_1e_1}|\phi\rangle_{A_2} \xrightarrow[\text{path 5}]{\substack{H^p,CPBS,NV_1,\\ CPBS,H^p}} & (\alpha_0|R\rangle|l_1I_1\rangle + \alpha_1|R\rangle|l_1E_1\rangle + \alpha_2|R\rangle|r_1I_1\rangle + \alpha_3|R\rangle|r_1E_1\rangle)_{A_1}|m^-\rangle_{e_1}|\phi\rangle_{A_2} \\
& +(\alpha_4|L\rangle|l_1I_1\rangle + \alpha_5|L\rangle|l_1E_1\rangle + \alpha_6|L\rangle|r_1I_1\rangle + \alpha_7|L\rangle|r_1E_1\rangle)_{A_1}|m^+\rangle_{e_1} \\
& \otimes[X^p(\beta_0|L\rangle|l_2I_2\rangle + \beta_4|R\rangle|l_2I_2\rangle) + \beta_1|R\rangle|l_2E_2\rangle + \beta_2|R\rangle|r_2I_2\rangle \\
& +\beta_3|R\rangle|r_2E_2\rangle + \beta_5|L\rangle|l_2E_2\rangle + \beta_6|L\rangle|r_2I_2\rangle + \beta_7|L\rangle|r_2E_2\rangle]_{A_2} \\
\xrightarrow[\text{path 6}]{\substack{H^p,CPBS,NV_1,\\ CPBS,H^p}} & (\alpha_0|R\rangle|l_1I_1\rangle + \alpha_1|R\rangle|l_1E_1\rangle + \alpha_2|R\rangle|r_1I_1\rangle + \alpha_3|R\rangle|r_1E_1\rangle)_{A_1}|m^-\rangle_{e_1}|\phi\rangle_{A_2} \\
& +(\alpha_4|L\rangle|l_1I_1\rangle + \alpha_5|L\rangle|l_1E_1\rangle + \alpha_6|L\rangle|r_1I_1\rangle + \alpha_7|L\rangle|r_1E_1\rangle)_{A_1}|m^+\rangle_{e_1} \\
& \otimes[X^p(\beta_0|L\rangle|l_2I_2\rangle + \beta_4|R\rangle|l_2I_2\rangle + \beta_1|R\rangle|l_2E_2\rangle + \beta_5|L\rangle|l_2E_2\rangle) \\
& +\beta_2|R\rangle|r_2I_2\rangle + \beta_3|R\rangle|r_2E_2\rangle + \beta_6|L\rangle|r_2I_2\rangle + \beta_7|L\rangle|r_2E_2\rangle]_{A_2} \\
\xrightarrow[\text{path 7}]{\substack{H^p,CPBS,NV_1,\\ CPBS,H^p}} & (\alpha_0|R\rangle|l_1I_1\rangle + \alpha_1|R\rangle|l_1E_1\rangle + \alpha_2|R\rangle|r_1I_1\rangle + \alpha_3|R\rangle|r_1E_1\rangle)_{A_1}|m^-\rangle_{e_1}|\phi\rangle_{A_2} \\
& +(\alpha_4|L\rangle|l_1I_1\rangle + \alpha_5|L\rangle|l_1E_1\rangle + \alpha_6|L\rangle|r_1I_1\rangle + \alpha_7|L\rangle|r_1E_1\rangle)_{A_1}|m^+\rangle_{e_1} \\
& \otimes[X^p(\beta_0|L\rangle|l_2I_2\rangle + \beta_4|R\rangle|l_2I_2\rangle + \beta_1|R\rangle|l_2E_2\rangle + \beta_5|L\rangle|l_2E_2\rangle \\
& +\beta_2|R\rangle|r_2I_2\rangle + \beta_6|L\rangle|r_2I_2\rangle) + \beta_3|R\rangle|r_2E_2\rangle + \beta_7|L\rangle|r_2E_2\rangle]_{A_2} \\
\xrightarrow[\text{path 8}]{\substack{H^p,CPBS,NV_1,\\ CPBS,H^p}} & (\alpha_0|R\rangle|l_1I_1\rangle + \alpha_1|R\rangle|l_1E_1\rangle + \alpha_2|R\rangle|r_1I_1\rangle + \alpha_3|R\rangle|r_1E_1\rangle)_{A_1}|m^-\rangle_{e_1}|\phi\rangle_{A_2} \\
& +(\alpha_4|L\rangle|l_1I_1\rangle + \alpha_5|L\rangle|l_1E_1\rangle + \alpha_6|L\rangle|r_1I_1\rangle + \alpha_7|L\rangle|r_1E_1\rangle)_{A_1}|m^+\rangle_{e_1} \\
& \otimes[X^p(\beta_0|L\rangle|l_2I_2\rangle + \beta_4|R\rangle|l_2I_2\rangle + \beta_1|R\rangle|l_2E_2\rangle + \beta_5|L\rangle|l_2E_2\rangle \\
& +\beta_2|R\rangle|r_2I_2\rangle + \beta_6|L\rangle|r_2I_2\rangle + \beta_3|R\rangle|r_2E_2\rangle + \beta_7|L\rangle|r_2E_2\rangle)]_{A_2}
\end{aligned}$$

which may collapse into

$$\begin{aligned}
&(\alpha_0|R\rangle|l_1I_1\rangle + \alpha_1|R\rangle|l_1E_1\rangle + \alpha_2|R\rangle|r_1I_1\rangle + \alpha_3|R\rangle|r_1E_1\rangle)_{A_1}|\phi\rangle_{A_2} \\
&+(\alpha_4|L\rangle|l_1I_1\rangle + \alpha_5|L\rangle|l_1E_1\rangle + \alpha_6|L\rangle|r_1I_1\rangle + \alpha_7|L\rangle|r_1E_1\rangle)_{A_1}(X^p|\phi\rangle_{A_2})
\end{aligned} \tag{4}$$

after measuring the NV center e_1 under the basis $\{|\pm\rangle\}$, where $X^p = |R\rangle\langle L| + |L\rangle\langle R|$ and $Z^p = |R\rangle\langle R| - |L\rangle\langle L|$ will be performed for the photon A_1 from each mode for the measurement outcome $|-\rangle_{e_1}$ of the NV center e_1.

Appendix B. CNOT gate on the spatial DoFs $\{l, r\}$ of two photons

First, from the Figure 3(b), the photon A_1 from the spatial mode r_1I_1 passes through CPBS, NV_2, X^p, NV_2, X^p, CPBS, sequentially. In detail, the pulse from the spatial mode r_1I_1 is separated from CPBS in the left. Its reflected pulse passes through the NV-cavity NV_2 firstly. And then the transmitted pulse passes through waveplate X^p, NV-cavity NV_2 and another waveplate X^p, sequentially. Now, the pulse output from the NV-cavity NV_2 and the pulse output from X^p will be combined at another CPBS simultaneously. Similar operations will be performed for the spatial mode r_1E_1. Generally, the photon A_1 and NV center e_2 will evolve as follows

$$\begin{aligned}
|\phi\rangle_{A_1}|+\rangle_{e_2} \xrightarrow[\text{mode } r_1I_1]{\substack{CPBS,NV_2,\\ X^p,NV_2,X^p,CPBS}} & (\alpha_2|R\rangle|r_1I_1\rangle + \alpha_6|L\rangle|r_1I_1\rangle)|-\rangle_{e_2} + (\alpha_0|R\rangle|l_1I_1\rangle + \alpha_4|L\rangle|l_1I_1\rangle \\
& +\alpha_1|R\rangle|l_1E_1\rangle + \alpha_3|R\rangle|r_1E_1\rangle + \alpha_5|L\rangle|l_1E_1\rangle + \alpha_7|L\rangle|r_1E_1\rangle)|+\rangle_{e_2} \\
\xrightarrow[\text{mode } r_1E_1]{\substack{CPBS,NV_2,\\ X^p,NV_2,X^p,CPBS}} & (\alpha_2|R\rangle|r_1I_1\rangle + \alpha_6|L\rangle|r_1I_1\rangle + \alpha_3|R\rangle|r_1E_1\rangle + \alpha_7|L\rangle|r_1E_1\rangle)|-\rangle_{e_2} \\
& +(\alpha_0|R\rangle|l_1I_1\rangle + \alpha_4|L\rangle|l_1I_1\rangle + \alpha_1|R\rangle|l_1E_1\rangle + \alpha_5|L\rangle|l_1E_1\rangle)|+\rangle_{e_2} \\
:= \quad & |\Psi_1\rangle_{A_1e_2} \qquad (5)
\end{aligned}$$

This is a controlled-Z gate(CZ_{se}) on the spatial DoF $\{|l\rangle, |r\rangle\}$ of the photon A_1 and the NV center e_2 [the NV center e_2 as the target qubit], i.e.,

$$CZ_{pe} := |l\rangle\langle l| \otimes I(|m^-\rangle\langle m^-| + |m^+\rangle\langle m^+|) + |r\rangle\langle r| \otimes (|m^-\rangle\langle m^-| - |m^+\rangle\langle m^+|) \qquad (6)$$

And then, after a H^a being performed on the NV center e_2, the photon A_2 evolves as follows

$$\begin{aligned}
& |\Psi_1\rangle_{A_1e_2}|\phi\rangle_{A_2} \\
\xrightarrow[\substack{\text{mode pairs}\\ (l_2I_2,r_2I_2);(l_2E_2,r_2E_2)}]{H^a;CBS;CBS} & [(\alpha_0|R\rangle|l_1I_1\rangle + \alpha_4|L\rangle|l_1I_1\rangle + \alpha_1|R\rangle|l_1E_1\rangle + \alpha_5|L\rangle|l_1E_1\rangle)_{A_1}|m^-\rangle_{e_2} \\
& +(\alpha_2|R\rangle|r_1I_1\rangle + \alpha_6|L\rangle|r_1I_1\rangle + \alpha_3|R\rangle|r_1E_1\rangle + \alpha_7|L\rangle|r_1E_1\rangle)_{A_1}|m^+\rangle_{e_2}] \\
& \otimes(\beta_0'|R\rangle|l_2I_2\rangle + \beta_2'|R\rangle|r_2I_2\rangle + \beta_4'|L\rangle|l_2I_2\rangle + \beta_6'|L\rangle|r_2I_2\rangle \\
& +\beta_1'|R\rangle|l_2E_2\rangle + \beta_3'|R\rangle|r_2E_2\rangle + \beta_5'|L\rangle|l_2E_2\rangle + \beta_7'|L\rangle|r_2E_2\rangle)_{A_2} \\
\xrightarrow[\text{mode } r_2I_2]{\substack{CPBS,NV_2,\\ X^p,NV_2,X^p,CPBS}} & (\alpha_0|R\rangle|l_1I_1\rangle + \alpha_4|L\rangle|l_1I_1\rangle + \alpha_1|R\rangle|l_1E_1\rangle + \alpha_5|L\rangle|l_1E_1\rangle)_{A_1}|m^-\rangle_{e_2} \\
& \otimes(\beta_0'|R\rangle|l_2I_2\rangle + \beta_2'|R\rangle|r_2I_2\rangle + \beta_4'|L\rangle|l_2I_2\rangle + \beta_6'|L\rangle|r_2I_2\rangle \\
& +\beta_1'|R\rangle|l_2E_2\rangle + \beta_3'|R\rangle|r_2E_2\rangle + \beta_5'|L\rangle|l_2E_2\rangle + \beta_7'|L\rangle|r_2E_2\rangle)_{A_2} \\
& +(\alpha_2|R\rangle|r_1I_1\rangle + \alpha_6|L\rangle|r_1I_1\rangle + \alpha_3|R\rangle|r_1E_1\rangle + \alpha_7|L\rangle|r_1E_1\rangle)_{A_1}|m^+\rangle_{e_1} \\
& \otimes[-(\beta_2'|R\rangle|r_2I_2\rangle + \beta_6'|L\rangle|r_2I_2\rangle) + \beta_0'|R\rangle|l_2I_2\rangle + \beta_4'|L\rangle|l_2I_2\rangle \\
& +\beta_1'|R\rangle|l_2E_2\rangle + \beta_3'|R\rangle|r_2E_2\rangle + \beta_5'|L\rangle|l_2E_2\rangle + \beta_7'|L\rangle|r_2E_2\rangle]_{A_2}
\end{aligned}$$

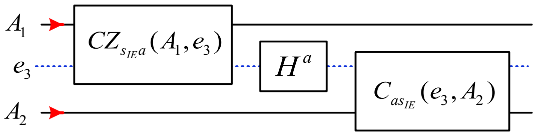

Figure S1| Schematic CNOT gate on the spatial DoFs $\{I, E\}$ of two photons. e_3 denotes an auxiliary NV center in the NV-cavity NV_3. The subcircuit $CZ_{s_{IE}a}$ for the spatial modes l_1E_1 and r_1E_1 of the photon A_1 is similar to $CZ_{s_{rl}a}$ in the Figure 3(b). The subcircuit $C_{as_{IE}}$ for the spatial mode pairs (l_1I_1, l_1E_1) and (r_1I_1, r_1E_1) of the photon A_1 is similar to $CZ_{as_{rl}}$ in the Figure 3(b).

$$
\begin{aligned}
\xrightarrow[\text{mode } r_2E_2]{\substack{CPBS, NV_2,\\ X^p, NV_2, X^p, CPBS}} \quad & (\alpha_0|R\rangle|l_1I_1\rangle + \alpha_4|L\rangle|l_1I_1\rangle + \alpha_1|R\rangle|l_1E_1\rangle + \alpha_5|L\rangle|l_1E_1\rangle)_{A_1}|m^-\rangle_{e_2} \\
& \otimes(\beta'_0|R\rangle|l_2I_2\rangle + \beta'_2|R\rangle|r_2I_2\rangle + \beta'_4|L\rangle|l_2I_2\rangle + \beta'_6|L\rangle|r_2I_2\rangle \\
& +\beta'_1|R\rangle|l_2E_2\rangle + \beta'_3|R\rangle|r_2E_2\rangle + \beta'_5|L\rangle|l_2E_2\rangle + \beta'_7|L\rangle|r_2E_2\rangle)_{A_2} \\
& +(\alpha_2|R\rangle|r_1I_1\rangle + \alpha_6|L\rangle|r_1I_1\rangle + \alpha_3|R\rangle|r_1E_1\rangle + \alpha_7|L\rangle|r_1E_1\rangle)_{A_1}|m^+\rangle_{e_2} \\
& \otimes[-(\beta'_2|R\rangle|r_2I_2\rangle + \beta'_6|L\rangle|r_2I_2\rangle + \beta'_3|R\rangle|r_2E_2\rangle + \beta'_7|L\rangle|r_2E_2\rangle) \\
& +\beta'_0|R\rangle|l_2I_2\rangle + \beta'_4|L\rangle|l_2I_2\rangle + \beta'_1|R\rangle|l_2E_2\rangle + \beta'_5|L\rangle|l_2E_2\rangle]_{A_2} \\
\xrightarrow[\substack{\text{mode pairs}\\ (l_2I_2, r_2I_2); (l_2E_2, r_2E_2)}]{CBS; CBS} \quad & (\alpha_0|R\rangle|l_1I_1\rangle + \alpha_4|L\rangle|l_1I_1\rangle + \alpha_1|R\rangle|l_1E_1\rangle + \alpha_5|L\rangle|l_1E_1\rangle)_{A_1}|m^-\rangle_{e_2}|\phi\rangle_{A_2} \\
& +(\alpha_2|R\rangle|r_1I_1\rangle + \alpha_6|L\rangle|r_1I_1\rangle + \alpha_3|R\rangle|r_1E_1\rangle + \alpha_7|L\rangle|r_1E_1\rangle)_{A_1}|m^+\rangle_{e_2} \\
& \otimes(\beta_0|R\rangle|r_2I_2\rangle + \beta_4|L\rangle|r_2I_2\rangle + \beta_1|R\rangle|r_2E_2\rangle + \beta_5|L\rangle|r_2E_2\rangle \\
& +\beta_2|R\rangle|l_2I_2\rangle + \beta_6|L\rangle|l_2I_2\rangle + \beta_3|R\rangle|l_2E_2\rangle + \beta_7|L\rangle|l_2E_2\rangle)_{A_2} \qquad (7)
\end{aligned}
$$

which may collapse into

$$
\begin{aligned}
& (\alpha_0|R\rangle|l_1I_1\rangle + \alpha_4|L\rangle|l_1I_1\rangle + \alpha_1|R\rangle|l_1E_1\rangle + \alpha_5|L\rangle|l_1E_1\rangle)_{A_1}|\phi\rangle_{A_2} \\
& +(\alpha_2|R\rangle|r_1I_1\rangle + \alpha_6|L\rangle|r_1I_1\rangle + \alpha_3|R\rangle|r_1E_1\rangle + \alpha_7|L\rangle|r_1E_1\rangle)_{A_1} \\
& \otimes(\beta_0|R\rangle|r_2I_2\rangle + \beta_4|L\rangle|r_2I_2\rangle + \beta_1|R\rangle|r_2E_2\rangle + \beta_5|L\rangle|r_2E_2\rangle \\
& +\beta_2|R\rangle|l_2I_2\rangle + \beta_6|L\rangle|l_2I_2\rangle + \beta_3|R\rangle|l_2E_2\rangle + \beta_7|L\rangle|l_2E_2\rangle)_{A_2} \qquad (8)
\end{aligned}
$$

after the measurement of the NV center e_2 under the basis $\{|\pm\rangle_{e_2}\}$, where Z^p will be performed for the photon A_1 from each mode r_1I_1 and r_1E_1 for the measurement outcome $|-\rangle_{e_2}$, $\beta'_0 = (\beta_0 + \beta_2)/\sqrt{2}$, $\beta'_2 = (\beta_0 - \beta_2)/\sqrt{2}$, $\beta'_4 = (\beta_4 + \beta_6)/\sqrt{2}$, $\beta'_6 = (\beta_4 - \beta_6)/\sqrt{2}$, $\beta'_1 = (\beta_1 + \beta_3)/\sqrt{2}$, $\beta'_3 = (\beta_1 - \beta_3)/\sqrt{2}$, $\beta'_5 = (\beta_5 + \beta_7)/\sqrt{2}$, and $\beta'_7 = (\beta_5 - \beta_7)/\sqrt{2}$.

Appendix C. CNOT gate on the spatial DoFs $\{I, E\}$ of two photons

From the Figure S1 in Supplementary Information and the Figure 3(b), a subcircuit $CZ_{s_{IE}a}$ is performed on the photon A_1 [from the spatial modes l_1E_1 and r_1E_1] and auxiliary NV center e_3 to

get

$$|\Psi_1'\rangle_{A_1e_3} := (\alpha_1|R\rangle|l_1E_1\rangle+\alpha_5|L\rangle|l_1E_1\rangle+\alpha_3|R\rangle|r_1E_1\rangle+\alpha_7|L\rangle|r_1E_1\rangle)|-\rangle_{e_3}$$
$$+(\alpha_2|R\rangle|r_1I_1\rangle+\alpha_6|L\rangle|r_1I_1\rangle+\alpha_0|R\rangle|l_1I_1\rangle+\alpha_4|L\rangle|l_1I_1\rangle)|+\rangle_{e_3} \quad (9)$$

This is a controlled-Z gate(CZ_{se}) on the spatial DoF $\{|I\rangle,|E\rangle\}$ of the photon A_1 and the NV center e_3 [the NV center e_3 as the target qubit], i.e.,

$$CZ_{pe} := |I\rangle\langle I|\otimes(|m^-\rangle\langle m^-|+|m^+\rangle\langle m^+|)+|E\rangle\langle E|\otimes(|m^-\rangle\langle m^-|-|m^+\rangle\langle m^+|) \quad (10)$$

And then, after a Hadamard transformation H^a being performed on the NV center e_3, the subcircuit $C_{as_{IE}}(e_3,A_2)$ is performed on the NV center e_3 and the photon A_2.

$$|\Psi_1'\rangle_{A_1e_3}|\phi\rangle_{A_2} \xrightarrow[C_{as_{IE}}(e_3,A_2)]{H^a} (\alpha_0|R\rangle|l_1I_1\rangle+\alpha_4|L\rangle|l_1I_1\rangle+\alpha_2|R\rangle|r_1I_1\rangle+\alpha_6|L\rangle|r_1I_1\rangle)_{A_1}|m^+\rangle_{e_3}|\phi\rangle_{A_2}$$
$$+(\alpha_1|R\rangle|l_1E_1\rangle+\alpha_5|L\rangle|l_1E_1\rangle+\alpha_3|R\rangle|r_1E_1\rangle+\alpha_7|L\rangle|r_1E_1\rangle)_{A_1}|m^-\rangle_{e_3}$$
$$\otimes(\beta_0|R\rangle|l_2E_2\rangle+\beta_4|L\rangle|l_2E_2\rangle+\beta_2|R\rangle|r_2E_2\rangle+\beta_6|L\rangle|r_2E_2\rangle$$
$$+\beta_1|R\rangle|l_2I_2\rangle+\beta_5|L\rangle|l_2I_2\rangle+\beta_3|R\rangle|r_2I_2\rangle+\beta_7|L\rangle|r_2I_2\rangle)_{A_2}$$
$$\xrightarrow{M_{e_3}^{\pm}} (\alpha_0|R\rangle|l_1I_1\rangle+\alpha_4|L\rangle|l_1I_1\rangle+\alpha_2|R\rangle|r_1I_1\rangle+\alpha_6|L\rangle|r_1I_1\rangle)_{A_1}|\phi\rangle_{A_2}$$
$$+(\alpha_1|R\rangle|l_1E_1\rangle+\alpha_5|L\rangle|l_1E_1\rangle+\alpha_3|R\rangle|r_1E_1\rangle+\alpha_7|L\rangle|r_1E_1\rangle)_{A_1}$$
$$\otimes(\beta_0|R\rangle|l_2E_2\rangle+\beta_4|L\rangle|l_2E_2\rangle+\beta_2|R\rangle|r_2E_2\rangle+\beta_6|L\rangle|r_2E_2\rangle$$
$$+\beta_1|R\rangle|l_2I_2\rangle+\beta_5|L\rangle|l_2I_2\rangle+\beta_3|R\rangle|r_2I_2\rangle+\beta_7|L\rangle|r_2I_2\rangle)_{A_2} \quad (11)$$

where $-I^P$ will be performed for the photon A_1 from each spatial mode l_1E_1 and r_1E_1 for the measurement outcome $|-\rangle_{e_3}$, $\beta_0=(\beta_0+\beta_1)/\sqrt{2}$, $\beta_1'=(\beta_0-\beta_1)/\sqrt{2}$, $\beta_2'=(\beta_2+\beta_3)/\sqrt{2}$, $\beta_3'=(\beta_2-\beta_3)/\sqrt{2}$, $\beta_4'=(\beta_4+\beta_5)/\sqrt{2}$, $\beta_5'=(\beta_4-\beta_5)/\sqrt{2}$, $\beta_6'=(\beta_6+\beta_7)/\sqrt{2}$, and $\beta_7'=(\beta_6-\beta_7)/\sqrt{2}$.

Appendix D. Hybrid CNOT gate on the polarization DoF of the photon A_1 and the spatial DoF $\{l,r\}$ of the photon A_2

From the Figure 4(a) and the Figure 3(a), after the photon A_1 from each spatial mode passes through CPBS, NV_1, CPBS, sequentially, the photon A_1 and the NV center e_1 will be changed into $|\Phi_1\rangle_{A_1e_1}$ as shown in the equation (3). And then, after a H^a being performed on the NV center e_1, the subcircuit $C_{as_{lr}}(e_1,A_2)$ is performed on the NV center e_1 and the photon A_2.

$$|\Phi_1\rangle_{A_1e_1}|\phi\rangle_{A_2} \xrightarrow[C_{as_{lr}}(e_1,A_2)]{H^a} (\alpha_0|R\rangle|l_1I_1\rangle+\alpha_1|R\rangle|l_1E_1\rangle+\alpha_2|R\rangle|r_1I_1\rangle+\alpha_3|R\rangle|r_1E_1\rangle)_{A_1}|m^-\rangle_{e_1}|\phi\rangle_{A_2}$$
$$+(\alpha_4|L\rangle|l_1I_1\rangle+\alpha_5|L\rangle|l_1E_1\rangle+\alpha_6|L\rangle|r_1I_1\rangle+\alpha_7|L\rangle|r_1E_1\rangle)_{A_1}|m^+\rangle_{e_1}$$
$$\otimes(\beta_0|R\rangle|r_2I_2\rangle+\beta_4|L\rangle|r_2I_2\rangle+\beta_1|R\rangle|r_2E_2\rangle+\beta_5|L\rangle|r_2E_2\rangle$$
$$+\beta_2|R\rangle|l_2I_2\rangle+\beta_6|L\rangle|l_2I_2\rangle+\beta_3|R\rangle|l_2E_2\rangle+\beta_7|L\rangle|l_2E_2\rangle]_{A_2}$$

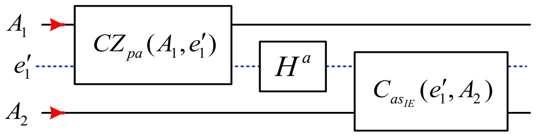

Figure S2| Schematic hybrid CNOT gate on the polarization DoF of the photon A_1 and the spatial DoF $\{I, E\}$ of the photon A_2. e_1' denotes an auxiliary NV center in the cavity NV_1'.

which may collapse into

$$
\begin{aligned}
&(\alpha_0|R\rangle|l_1I_1\rangle + \alpha_1|R\rangle|l_1E_1\rangle + \alpha_2|R\rangle|r_1I_1\rangle + \alpha_3|R\rangle|r_1E_1\rangle)_{A_1}|\phi\rangle_{A_2}\\
&+(\alpha_4|L\rangle|l_1I_1\rangle + \alpha_5|L\rangle|l_1E_1\rangle + \alpha_6|L\rangle|r_1I_1\rangle + \alpha_7|L\rangle|r_1E_1\rangle)_{A_1}\\
&\otimes(\beta_0|R\rangle|r_2I_2\rangle + \beta_4|L\rangle|r_2I_2\rangle + \beta_1|R\rangle|r_2E_2\rangle + \beta_5|L\rangle|r_2E_2\rangle\\
&+\beta_2|R\rangle|l_2I_2\rangle + \beta_6|L\rangle|l_2I_2\rangle + \beta_3|R\rangle|l_2E_2\rangle + \beta_7|L\rangle|l_2E_2\rangle)_{A_2} \qquad (12)
\end{aligned}
$$

after the measurement of the NV center e_1 under the basis $\{|\pm\rangle_{e_1}\}$, where Z^p will be performed for the photon A_1 from each mode for the measurement outcome $|-\rangle_{e_1}$, $\beta_0' = (\beta_0 + \beta_2)/\sqrt{2}$, $\beta_2' = (\beta_0 - \beta_2)/\sqrt{2}$, $\beta_1' = (\beta_1 + \beta_3)/\sqrt{2}$, $\beta_3' = (\beta_1 - \beta_3)/\sqrt{2}$, $\beta_4' = (\beta_4 + \beta_6)/\sqrt{2}$, $\beta_6' = (\beta_4 - \beta_6)/\sqrt{2}$, $\beta_5' = (\beta_5 + \beta_7)/\sqrt{2}$, and $\beta_7' = (\beta_5 - \beta_7)/\sqrt{2}$.

Appendix E. Hybrid CNOT gate on polarization DoF of the photon A_1 and spatial DoF $\{I, E\}$ of the photon A_2

From the Figure S2 in Supplementary Information, after the photon A_1 from each spatial mode passes through CPBS, NV_1', CPBS, sequentially, the photon A_1 and the NV center e_1' will be changed into $|\Phi_1\rangle_{A_1e_1'}$ as shown in the equation (3). And then, after a H^a being performed on the NV center e_1', the subcircuit $C_{as_{IE}}(e_1', A_2)$ is performed on the NV center e_1' and the photon A_2.

$$
\begin{aligned}
|\Phi_1\rangle_{A_1e_1'}|\phi\rangle_{A_2} \xrightarrow[C_{as_{IE}}(e_1',A_2)]{H^a} &(\alpha_0|R\rangle|l_1I_1\rangle + \alpha_1|R\rangle|l_1E_1\rangle + \alpha_2|R\rangle|r_1I_1\rangle + \alpha_3|R\rangle|r_1E_1\rangle)_{A_1}|m^-\rangle_{e_1'}|\phi\rangle_{A_2}\\
&+(\alpha_4|L\rangle|l_1I_1\rangle + \alpha_5|L\rangle|l_1E_1\rangle + \alpha_6|L\rangle|r_1I_1\rangle + \alpha_7|L\rangle|r_1E_1\rangle)_{A_1}|m^+\rangle_{e_1'}\\
&\otimes(\beta_0|R\rangle|l_2E_2\rangle + \beta_4|L\rangle|l_2E_2\rangle + \beta_2|R\rangle|r_2E_2\rangle + \beta_6|L\rangle|r_2E_2\rangle\\
&+\beta_1|R\rangle|l_2I_2\rangle + \beta_5|L\rangle|l_2I_2\rangle + \beta_3|R\rangle|r_2I_2\rangle + \beta_7|L\rangle|r_2I_2\rangle)_{A_2}\\
\xrightarrow{M^{\pm}_{e_1'}} &(\alpha_0|R\rangle|l_1I_1\rangle + \alpha_1|R\rangle|l_1E_1\rangle + \alpha_2|R\rangle|r_1I_1\rangle + \alpha_3|R\rangle|r_1E_1\rangle)_{A_1}|\phi\rangle_{A_2}\\
&+(\alpha_4|L\rangle|l_1I_1\rangle + \alpha_5|L\rangle|l_1E_1\rangle + \alpha_6|L\rangle|r_1I_1\rangle + \alpha_7|L\rangle|r_1E_1\rangle)_{A_1}\\
&\otimes(\beta_0|R\rangle|l_2E_2\rangle + \beta_4|L\rangle|l_2E_2\rangle + \beta_2|R\rangle|r_2E_2\rangle + \beta_6|L\rangle|r_2E_2\rangle\\
&+\beta_1|R\rangle|l_2I_2\rangle + \beta_5|L\rangle|l_2I_2\rangle + \beta_3|R\rangle|r_2I_2\rangle + \beta_7|L\rangle|r_2I_2\rangle)_{A_2} \qquad (13)
\end{aligned}
$$

where $M^{\pm}_{e_1'}$ denotes the measurement of the NV center e_1' under the basis $\{|\pm\rangle_{e_1'}\}$, and $-I^p = -|R\rangle\langle R| - |L\rangle\langle L|$ will be performed for the photon A_1 from each spatial mode $|l_1E_1\rangle$ and $|r_1E_1\rangle$ for

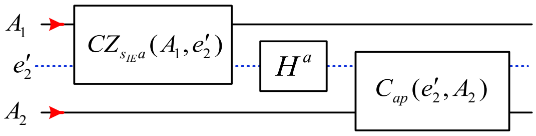

Figure S3| Schematic hybrid CNOT gate on the spatial DoF $\{I, E\}$ of the photon A_1 and the polarization DoF of the photon A_2. e_2' denotes an auxiliary NV center charged in the cavity NV_2'.

the measurement outcome $|-\rangle_{e_1'}$. Here, $\beta_0' = (\beta_0+\beta_1)/\sqrt{2}$, $\beta_1' = (\beta_0-\beta_1)/\sqrt{2}$, $\beta_2' = (\beta_2+\beta_3)/\sqrt{2}$, $\beta_3' = (\beta_2-\beta_3)/\sqrt{2}$, $\beta_4' = (\beta_4+\beta_5)/\sqrt{2}$, $\beta_5' = (\beta_4-\beta_5)/\sqrt{2}$, $\beta_6' = (\beta_6+\beta_7)/\sqrt{2}$, and $\beta_7' = (\beta_6-\beta_7)/\sqrt{2}$.

Appendix F. Hybrid CNOT gate on the spatial DoF $\{l, r\}$ of the photon A_1 and the polarization DoF of the photon A_2

From the Figure 4(b) the photon A_1 from the spatial mode r_1I_1 passes through CPBS, NV_2, X^p, NV_2, X^p, CPBS, sequentially. Similar operations will be performed for the photon A_1 from the spatial mode r_1E_1. After these operations, the photon A_1 and NV center e_1 are changed into $|\Psi_1\rangle$ as shown in the equation (5). After a H^a performed on the NV center e_1, the photon A_2 from each mode will pass through H^p, CPBS, NV_2, CPBS, H^p, sequentially to complete the subcircuit $C_{ap}(e_1, A_2)$. In detail,

$$\begin{aligned}|\Psi_1\rangle_{A_1e_2}|\phi\rangle_{A_2} \xrightarrow[C_{ap}(e_1,A_2)]{H^p} & (\alpha_0|R\rangle|l_1I_1\rangle + \alpha_4|L\rangle|l_1I_1\rangle + \alpha_1|R\rangle|l_1E_1\rangle + \alpha_5|L\rangle|l_1E_1\rangle)_{A_1}|\phi\rangle_{A_2} \\ & +(\alpha_2|R\rangle|r_1I_1\rangle + \alpha_6|L\rangle|r_1I_1\rangle + \alpha_3|R\rangle|r_1E_1\rangle + \alpha_7|L\rangle|r_1E_1\rangle)_{A_1} \\ & \otimes(\beta_4|R\rangle|l_2I_2\rangle + \beta_0|L\rangle|l_2I_2\rangle + \beta_2|R\rangle|r_2I_2\rangle + \beta_6|L\rangle|r_2I_2\rangle \\ & +\beta_5|R\rangle|l_2E_2\rangle + \beta_1|L\rangle|l_2E_2\rangle + \beta_3|R\rangle|r_2E_2\rangle + \beta_7|L\rangle|r_2E_2\rangle)_{A_2} \quad (14)\end{aligned}$$

after the measurement of the NV center e_2 under the basis $\{|\pm\rangle_{e_2}\}$, where $-I^p$ will be performed for the photon A_1 from each mode r_1I_1 and r_1E_1 for the measurement outcome $|-\rangle_{e_2}$.

Appendix G. Hybrid CNOT gate on the spatial DoF $\{I, E\}$ of the photon A_1 and the polarization DoF of the photon A_2

From the Figure S3 in Supplementary Information, the photon A_1 from the spatial mode l_1E_1 and r_1E_1 passes through CPBS, NV_2', X^p, NV_2', X^p, CPBS, sequentially. After these operations, the photon A_1 and NV center e_2' are changed into $|\Psi_1\rangle$ as shown in the equation (9). After a H^a performed on the NV center e_2', the photon A_2 from each mode will pass through H^p, CPBS, NV_2',

CPBS, H^p, sequentially. In detail,

$$
\begin{aligned}
|\Psi'_1\rangle_{A_1e'_2}|\phi\rangle_{A_2} \xrightarrow[C_{ap}(e'_2,A_2)]{H^p} & (\alpha_0|R\rangle|l_1I_1\rangle+\alpha_4|L\rangle|l_1I_1\rangle+\alpha_2|R\rangle|r_1I_1\rangle+\alpha_6|L\rangle|r_1I_1\rangle)_{A_1}|m^-\rangle_{e'_2}|\phi\rangle_{A_2}\\
&+(\alpha_1|R\rangle|l_1E_1\rangle+\alpha_5|L\rangle|l_1E_1\rangle+\alpha_3|R\rangle|r_1E_1\rangle+\alpha_7|L\rangle|r_1E_1\rangle)_{A_1}|m^+\rangle_{e'_2}\\
&\otimes(\beta_4|R\rangle|l_2I_2\rangle+\beta_0|L\rangle|l_2I_2\rangle+\beta_2|R\rangle|r_2I_2\rangle+\beta_6|L\rangle|r_2I_2\rangle\\
&+\beta_5|R\rangle|l_2E_2\rangle+\beta_1|L\rangle|l_2E_2\rangle+\beta_7|R\rangle|r_2E_2\rangle+\beta_3|L\rangle|r_2E_2\rangle)_{A_2}
\end{aligned}
$$

which may collapse into

$$
\begin{aligned}
&(\alpha_0|R\rangle|l_1I_1\rangle+\alpha_4|L\rangle|l_1I_1\rangle+\alpha_2|R\rangle|r_1I_1\rangle+\alpha_6|L\rangle|r_1I_1\rangle)_{A_1}|\phi\rangle_{A_2}\\
&+(\alpha_1|R\rangle|l_1E_1\rangle+\alpha_5|L\rangle|l_1E_1\rangle+\alpha_3|R\rangle|r_1E_1\rangle+\alpha_7|L\rangle|r_1E_1\rangle)_{A_1}\\
&\otimes(\beta_4|R\rangle|l_2I_2\rangle+\beta_0|L\rangle|l_2I_2\rangle+\beta_2|R\rangle|r_2I_2\rangle+\beta_6|L\rangle|r_2I_2\rangle\\
&+\beta_5|R\rangle|l_2E_2\rangle+\beta_1|L\rangle|l_2E_2\rangle+\beta_3|R\rangle|r_2E_2\rangle+\beta_7|L\rangle|r_2E_2\rangle)_{A_2}
\end{aligned} \tag{15}
$$

after measuring the NV center e'_2 under the basis $\{|\pm\rangle_{e'_2}\}$, where $-I^p$ will be performed for the photon A_1 from each mode l_1E_1 and r_1E_1 for the measurement outcome $|-\rangle_{e'_2}$.

Appendix H. Hybrid CNOT gate on the spatial DoF $\{l, r\}$ of the photon A_1 and the spatial DoF $\{I, E\}$ of the photon A_2

First, from the Figure 4(c) the photon A_1 from the spatial modes r_1I_1 and r_1E_1 pass through CPBS, NV_3, X^p, NV_3, X^p, CPBS, sequentially. After these operations, the photon A_1 and NV center e_3 are changed into $|\Psi_1\rangle$ as shown in the equation (5). After a Hadamard operation H^a performed on the NV center e_3, the subcircuit $C_{as_{IE}}(e_3, A_2)$ is performed on the NV center e_3 and the photon A_2.

$$
\begin{aligned}
|\Psi_1\rangle_{A_1e_3}|\phi\rangle_{A_2} \xrightarrow[C_{as_{IE}}(e_3,A_2)]{H^a} & (\alpha_0|R\rangle|l_1I_1\rangle+\alpha_4|L\rangle|l_1I_1\rangle+\alpha_1|R\rangle|l_1E_1\rangle+\alpha_5|L\rangle|l_1E_1\rangle)_{A_1}|m^-\rangle_{e_3}|\phi\rangle_{A_2}\\
&+(\alpha_2|R\rangle|r_1I_1\rangle+\alpha_6|L\rangle|r_1I_1\rangle+\alpha_3|R\rangle|r_1E_1\rangle+\alpha_7|L\rangle|r_1E_1\rangle)_{A_1}|m^+\rangle_{e_3}\\
&\otimes(\beta_0|R\rangle|l_2E_2\rangle+\beta_4|L\rangle|l_2E_2\rangle+\beta_2|R\rangle|r_2E_2\rangle+\beta_6|L\rangle|r_2E_2\rangle\\
&+\beta_1|R\rangle|l_2I_2\rangle+\beta_5|L\rangle|l_2I_2\rangle+\beta_3|R\rangle|r_2I_2\rangle+\beta_7|L\rangle|r_2I_2\rangle)_{A_2}\\
\xrightarrow{M^{\pm}_{e_3}} & (\alpha_0|R\rangle|l_1I_1\rangle+\alpha_4|L\rangle|l_1I_1\rangle+\alpha_1|R\rangle|l_1E_1\rangle+\alpha_5|L\rangle|l_1E_1\rangle)_{A_1}|\phi\rangle_{A_2}\\
&+(\alpha_2|R\rangle|r_1I_1\rangle+\alpha_6|L\rangle|r_1I_1\rangle+\alpha_3|R\rangle|r_1E_1\rangle+\alpha_7|L\rangle|r_1E_1\rangle)_{A_1}\\
&\otimes(\beta_0|R\rangle|l_2E_2\rangle+\beta_4|L\rangle|l_2E_2\rangle+\beta_2|R\rangle|r_2E_2\rangle+\beta_6|L\rangle|r_2E_2\rangle\\
&+\beta_1|R\rangle|l_2I_2\rangle+\beta_5|L\rangle|l_2I_2\rangle+\beta_3|R\rangle|r_2I_2\rangle+\beta_7|L\rangle|r_2I_2\rangle)_{A_2}
\end{aligned} \tag{16}
$$

where two phase flips $-I^p$s will be performed for the photon A_1 from the spatial modes l_1E_1 and r_1E_1 for the measurement outcome $|-\rangle_{e_3}$, $\beta'_0 = (\beta_0+\beta_1)/\sqrt{2}$, $\beta'_1 = (\beta_0-\beta_1)/\sqrt{2}$, $\beta'_2 = (\beta_2+\beta_3)/\sqrt{2}$, $\beta'_3 = (\beta_2-\beta_3)/\sqrt{2}$, $\beta'_4 = (\beta_4+\beta_5)/\sqrt{2}$, $\beta'_5 = (\beta_4-\beta_5)/\sqrt{2}$, $\beta'_6 = (\beta_6+\beta_7)/\sqrt{2}$, and $\beta'_7 = (\beta_6-\beta_7)/\sqrt{2}$.

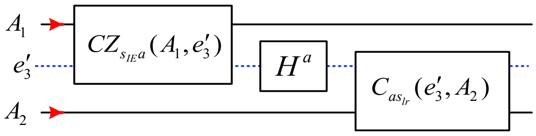

Figure S4| Schematic hybrid CNOT gate on the spatial DoF $\{I, E\}$ of the photon A_1 and the spatial DoF $\{l, r\}$ of the photon A_2. e'_3 denotes an auxiliary NV center charged in the cavity NV'_3.

Appendix I. Hybrid CNOT gate on the spatial DoF $\{I, E\}$ of the photon A_1 and the spatial DoF $\{l, r\}$ of the photon A_2

From the Figure S4 in Supplementary Information, the photon A_1 from the spatial modes l_1E_1 and r_1E_1 pass through CPBS, NV'_3, X^p, NV'_3, X^p, CPBS, sequentially. After these operations, the photon A_1 and NV center e'_3 are changed into $|\Psi'_1\rangle$ as shown in the equation (9). After a H^a performed on the NV center e'_3, the subcircuit $C_{as_{lr}}(e'_3, A_2)$ is performed on the photon A_2 and the NV center e'_3.

$$
\begin{aligned}
|\Psi'_1\rangle_{A_1e'_3}|\phi\rangle_{A_2} \xrightarrow[C_{as_{lr}}(e'_3,A_2)]{H^a} \; & (\alpha_0|R\rangle|l_1I_1\rangle + \alpha_4|L\rangle|l_1I_1\rangle + \alpha_2|R\rangle|r_1I_1\rangle + \alpha_6|L\rangle|r_1I_1\rangle)_{A_1}|m^-\rangle_{e'_3}|\phi\rangle_{A_2} \\
& + (\alpha_1|R\rangle|l_1E_1\rangle + \alpha_5|L\rangle|l_1E_1\rangle + \alpha_3|R\rangle|r_1E_1\rangle + \alpha_7|L\rangle|r_1E_1\rangle)_{A_1}|m^+\rangle_{e'_3} \\
& \otimes(\beta_0|R\rangle|r_2I_2\rangle + \beta_1|R\rangle|r_2E_2\rangle + \beta_4|L\rangle|r_2I_2\rangle + \beta_5|L\rangle|r_2E_2\rangle \\
& + \beta_2|R\rangle|l_2I_2\rangle + \beta_3|R\rangle|l_2E_2\rangle + \beta_6|L\rangle|l_2I_2\rangle + \beta_7|L\rangle|l_2E_2\rangle)_{A_2} \qquad (17)
\end{aligned}
$$

which may collapse into

$$
\begin{aligned}
& (\alpha_0|R\rangle|l_1I_1\rangle + \alpha_4|L\rangle|l_1I_1\rangle + \alpha_2|R\rangle|r_1I_1\rangle + \alpha_6|L\rangle|r_1I_1\rangle)_{A_1}|\phi\rangle_{A_2} \\
& + (\alpha_1|R\rangle|l_1E_1\rangle + \alpha_5|L\rangle|l_1E_1\rangle + \alpha_3|R\rangle|r_1E_1\rangle + \alpha_7|L\rangle|r_1E_1\rangle)_{A_1} \\
& \otimes(\beta_0|R\rangle|r_2I_2\rangle + \beta_4|L\rangle|r_2I_2\rangle + \beta_1|R\rangle|r_2E_2\rangle + \beta_5|L\rangle|r_2E_2\rangle \\
& + \beta_2|R\rangle|l_2I_2\rangle + \beta_6|L\rangle|l_2I_2\rangle + \beta_3|R\rangle|l_2E_2\rangle + \beta_7|L\rangle|l_2E_2\rangle)_{A_2} \qquad (18)
\end{aligned}
$$

after the measurement of the NV center e'_3 under the basis $\{|\pm\rangle_{e'_3}\}$, where Z^p will be performed for the photon A_1 from each mode r_1I_1 and r_1E_1 for the measurement outcome $|-\rangle_{e'_3}$, $\beta'_0 = (\beta_0 + \beta_2)/\sqrt{2}$, $\beta'_2 = (\beta_0 - \beta_2)/\sqrt{2}$, $\beta'_1 = (\beta_1 + \beta_3)/\sqrt{2}$, $\beta'_3 = (\beta_1 - \beta_3)/\sqrt{2}$, $\beta'_4 = (\beta_4 + \beta_6)/\sqrt{2}$, $\beta'_6 = (\beta_4 - \beta_6)/\sqrt{2}$, $\beta'_5 = (\beta_5 + \beta_7)/\sqrt{2}$, and $\beta'_7 = (\beta_5 - \beta_7)/\sqrt{2}$.

Appendix J. Hybrid CNOT gate on the polarization DoF and the spatial DoF $\{l, r\}$ of one photon

From the Figure 5, after the photon A_1 from each spatial mode passes through CPBS, NV_1, CPBS, sequentially, the photon A_1 and NV center e_1 will be changed into $|\Phi_1\rangle_{A_1e_1}$ as shown in the equation

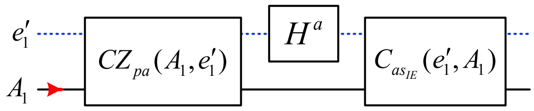

Figure S5| Schematic CNOT gate on the polarization DoF and the spatial DoF $\{I, E\}$ of the photon A_1. e_1' denotes an auxiliary NV center charged in the cavity NV_1'.

(3). And then, after a H^a performed on the NV center e_1, the subcircuit $C_{as_{lr}}(e_1, A_1)$ is performed on the NV center e_1 and photon A_1.

$$\begin{aligned}|\Phi_1\rangle_{A_1 e_1} \xrightarrow[C_{as_{lr}}(e_1,A_1)]{H^a} & (\alpha_0|R\rangle|l_1 I_1\rangle + \alpha_1|R\rangle|l_1 E_1\rangle + \alpha_2|R\rangle|r_1 I_1\rangle + \alpha_3|R\rangle|r_1 E_1\rangle)_{A_1}|m^-\rangle_{e_1} \\ & +(\alpha_6|L\rangle|l_1 I_1\rangle + \alpha_4|L\rangle|r_1 I_1\rangle + \alpha_7|L\rangle|l_1 E_1\rangle + \alpha_5|L\rangle|r_1 E_1\rangle)_{A_1}|m^+\rangle_{e_1}\end{aligned}$$

which may collapse into

$$\begin{aligned}&\alpha_0|R\rangle|l_1 I_1\rangle + \alpha_1|R\rangle|l_1 E_1\rangle + \alpha_2|R\rangle|r_1 I_1\rangle + \alpha_3|R\rangle|r_1 E_1\rangle \\ &+\alpha_4|L\rangle|r_1 I_1\rangle + \alpha_5|L\rangle|r_1 E_1\rangle + \alpha_6|L\rangle|l_1 I_1\rangle + \alpha_7|L\rangle|l_1 E_1\rangle\end{aligned} \tag{19}$$

after the measurement of the NV center e_1 under the basis $\{|\pm\rangle_{e_1}\}$, where Z^p will be performed for the photon A_1 from each mode for the measurement outcome $|-\rangle_{e_1}$, $\alpha_0' = (\alpha_0 + \alpha_2)/\sqrt{2}$, $\alpha_2' = (\alpha_0 - \alpha_2)/\sqrt{2}$, $\alpha_1' = (\alpha_1 + \alpha_3)/\sqrt{2}$, $\alpha_3' = (\alpha_1 - \alpha_3)/\sqrt{2}$, $\beta_4' = (\alpha_4 + \alpha_6)/\sqrt{2}$, $\alpha_6' = (\alpha_4 - \alpha_6)/\sqrt{2}$, $\alpha_5' = (\alpha_5 + \alpha_7)/\sqrt{2}$, and $\alpha_7' = (\alpha_5 - \alpha_7)/\sqrt{2}$.

Appendix K. Hybrid CNOT gate on the polarization DoF and the spatial DoF $\{I, E\}$ of one photon

From the Figure S5 in Supplementary Information, after the photon A_1 from each spatial mode passes through CPBS, NV_1', CPBS, sequentially, the photon A_1 and NV center e_1' will be changed into $|\Phi_1\rangle_{A_1 e_1'}$ as shown in the equation (3). And then, after a H^a performed on the NV center e_1', the subcircuit $C_{as_{IE}}(e_1, A_1)$ is performed on the NV center e_1' and the photon A_1.

$$\begin{aligned}|\Phi_1\rangle_{A_1 e_1'} \xrightarrow[C_{as_{IE}}(e_1,A_1)]{H^a} & (\alpha_0|R\rangle|l_1 I_1\rangle + \alpha_1|R\rangle|l_1 E_1\rangle + \alpha_2|R\rangle|r_1 I_1\rangle + \alpha_3|R\rangle|r_1 E_1\rangle)_{A_1}|m^-\rangle_{e_1'} \\ & +(\alpha_5|L\rangle|l_1 I_1\rangle + \alpha_4|L\rangle|l_1 E_1\rangle + \alpha_7|L\rangle|r_1 I_1\rangle + \alpha_6|L\rangle|r_1 E_1\rangle)_{A_1}|m^+\rangle_{e_1'}\end{aligned} \tag{20}$$

which may collapse into

$$\begin{aligned}&\alpha_0|R\rangle|l_1 I_1\rangle + \alpha_1|R\rangle|l_1 E_1\rangle + \alpha_2|R\rangle|r_1 I_1\rangle + \alpha_3|R\rangle|r_1 E_1\rangle \\ &+\alpha_4|L\rangle|l_1 E_1\rangle + \alpha_5|L\rangle|l_1 I_1\rangle + \alpha_6|L\rangle|r_1 E_1\rangle + \alpha_7|L\rangle|r_1 I_1\rangle\end{aligned} \tag{21}$$

after the measurement of the NV center e_1' under the basis $\{|\pm\rangle_{e_1'}\}$, where Z^p will be performed for the photon A_1 from each mode for the measurement outcome $|-\rangle_{e_1'}$, $\alpha_0' = (\alpha_0 + \alpha_1)/\sqrt{2}$,

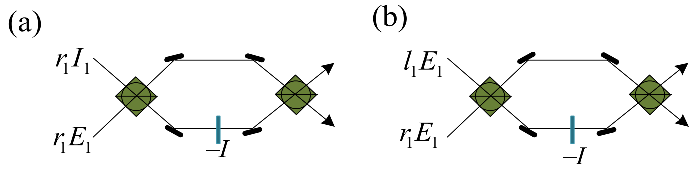

Figure S6| Hybrid CNOT gate on two spatial DoFs of the photon A_1 (a) Schematic hybrid CNOT gate on the spatial DoF $\{l, r\}$ and the spatial DoF $\{I, E\}$ of the photon A_1. (b) Schematic CNOT gate on the spatial DoF $\{I, E\}$ and the spatial DoF $\{l, r\}$ of the photon A_1.

$\alpha'_1 = (\alpha_0-\alpha_1)/\sqrt{2}$, $\alpha'_2 = (\alpha_2+\alpha_3)/\sqrt{2}$, $\alpha'_3 = (\alpha_2-\alpha_3)/\sqrt{2}$, $\beta'_4 = (\alpha_4+\alpha_5)/\sqrt{2}$, $\alpha'_5 = (\alpha_4-\alpha_5)/\sqrt{2}$, $\alpha'_6 = (\alpha_6+\alpha_7)/\sqrt{2}$, and $\alpha'_7 = (\alpha_6-\alpha_7)/\sqrt{2}$.

Appendix L. CNOT gate on two spatial DoFs of one photon

Figure S6(a) presents a schematic hybrid CNOT gate on the spatial DoF $\{l, r\}$ and the spatial DoF $\{I, E\}$ of the photon A_1. Here, the photon A_1 from the spatial modes r_1I_1 and r_1E_1 pass through CBS, $-I$, CBS, sequentially. The photon A_1 evolves as follows

$$
\begin{aligned}
|\phi_1\rangle_{A_1} \xrightarrow[\text{mode pair } (r_1I_1, r_1E_1)]{CBS} & \alpha_0|R\rangle|l_1I_1\rangle + \alpha_1|R\rangle|l_1E_1\rangle + \alpha_4|L\rangle|l_1I_1\rangle + \alpha_5|L\rangle|l_1E_1\rangle \\
& +\alpha'_2|R\rangle|r_1I_1\rangle + \alpha'_3|R\rangle|r_1E_1\rangle + \alpha'_6|L\rangle|r_1I_1\rangle + \alpha'_7|L\rangle|r_1E_1\rangle \\
\xrightarrow[\text{mode } r_1E_1]{-I} & \alpha_0|R\rangle|l_1I_1\rangle + \alpha_1|R\rangle|l_1E_1\rangle + \alpha_4|L\rangle|l_1I_1\rangle + \alpha_5|L\rangle|l_1E_1\rangle \\
& +\alpha'_2|R\rangle|r_1I_1\rangle - \alpha'_3|R\rangle|r_1E_1\rangle + \alpha'_6|L\rangle|r_1I_1\rangle - \alpha'_7|L\rangle|r_1E_1\rangle \\
\xrightarrow[\text{mode pair } (r_1I_1, r_1E_1)]{CBS} & \alpha_0|R\rangle|l_1I_1\rangle + \alpha_1|R\rangle|l_1E_1\rangle + \alpha_4|L\rangle|l_1I_1\rangle + \alpha_5|L\rangle|l_1E_1\rangle \\
& +\alpha_3|R\rangle|r_1I_1\rangle + \alpha_2|R\rangle|r_1E_1\rangle + \alpha_7|L\rangle|r_1I_1\rangle + \alpha_6|L\rangle|r_1E_1\rangle
\end{aligned} \quad (22)
$$

where $\alpha'_2 = (\alpha_2+\alpha_3)/\sqrt{2}$, $\alpha'_3 = (\alpha_2-\alpha_3)/\sqrt{2}$, $\alpha'_6 = (\alpha_6+\alpha_7)/\sqrt{2}$, and $\alpha'_7 = (\alpha_6-\alpha_7)/\sqrt{2}$.

Figure S6(b) presents a schematic hybrid CNOT gate on the spatial DoF $\{I, E\}$ and the spatial DoF $\{l, r\}$ of the photon A_1. Here, the photon A_1 from the spatial modes l_1E_1 and r_1E_1 pass through CBS, $-I$, CBS, sequentially. The photon A_1 evolves as follows

$$
\begin{aligned}
|\phi_1\rangle_{A_1} \xrightarrow[\text{mode pair } (l_1E_1, r_1E_1)]{CBS} & \alpha_0|R\rangle|l_1I_1\rangle + \alpha_4|L\rangle|l_1I_1\rangle + \alpha_2|R\rangle|r_1I_1\rangle + \alpha_6|L\rangle|r_1I_1\rangle \\
& +\alpha'_1|R\rangle|l_1E_1\rangle + \alpha'_3|R\rangle|r_1E_1\rangle + \alpha'_5|L\rangle|l_1E_1\rangle + \alpha'_7|L\rangle|r_1E_1\rangle \\
\xrightarrow[\text{mode } r_1E_1]{-I} & \alpha_0|R\rangle|l_1I_1\rangle + \alpha_4|L\rangle|l_1I_1\rangle + \alpha_2|R\rangle|r_1I_1\rangle + \alpha_6|L\rangle|r_1I_1\rangle \\
& +\alpha'_1|R\rangle|l_1E_1\rangle - \alpha'_3|R\rangle|r_1E_1\rangle + \alpha'_5|L\rangle|l_1E_1\rangle - \alpha'_7|L\rangle|r_1E_1\rangle \\
\xrightarrow[\text{mode pair } (r_1I_1, r_1E_1)]{CBS} & \alpha_0|R\rangle|l_1I_1\rangle + \alpha_4|L\rangle|l_1I_1\rangle + \alpha_2|R\rangle|r_1I_1\rangle + \alpha_6|L\rangle|r_1I_1\rangle \\
& +\alpha_3|R\rangle|l_1E_1\rangle + \alpha_1|R\rangle|r_1E_1\rangle + \alpha_7|L\rangle|l_1E_1\rangle + \alpha_5|L\rangle|r_1E_1\rangle
\end{aligned} \quad (23)
$$

where $\alpha_1' = (\alpha_1 + \alpha_3)/\sqrt{2}$, $\alpha_3' = (\alpha_1 - \alpha_3)/\sqrt{2}$, $\alpha_5' = (\alpha_5 + \alpha_7)/\sqrt{2}$, and $\alpha_7' = (\alpha_5 - \alpha_7)/\sqrt{2}$.
